# Supplementary material for: Charity financial support to motor neuron disease (MND) patients in Greater London: the impact of patients’ socioeconomic status—a cross-sectional study
Source: BMJ Open. 2019 Feb 12;9(2):e022462. doi: 10.1136/bmjopen-2018-022462 (PMC6377508; doi:10.1136/bmjopen-2018-022462)
Supplement: Author's manuscript [file bmjopen-2018-022462.draft_revisions.pdf]

BMJ Open

BMJ Open is committed to open peer review. As part of this commitment we make the peer review history of every article we publish publicly available.

When an article is published we post the peer reviewers' comments and the authors' responses online. We also post the versions of the paper that were used during peer review. These are the versions that the peer review comments apply to.

The versions of the paper that follow are the versions that were submitted during the peer review process. They are not the versions of record or the final published versions. They should not be cited or distributed as the published version of this manuscript.

BMJ Open is an open access journal and the full, final, typeset and author-corrected version of record of the manuscript is available on our site with no access controls, subscription charges or pay-per-view fees (http://bmjopen.bmj.com).

If you have any questions on BMJ Open's open peer review process please email info.bmjopen@bmj.com

BMJ Open

Is charity support distributed equally or fairly? The case of Motor Neurone Disease (MND) Association and its work in Greater London

Journal:	*BMJ Open*
Manuscript ID	bmjopen-2018-022462
Article Type:	Research
Date Submitted by the Author:	05-Mar-2018
Complete List of Authors:	Giouleka, Anna; Barts and The London School of Medicine and Dentistry, CPCPH<br>Manning, Alison; Motor Neurone Disease Association<br>Smith, Dianna; University of Southampton, Geography & Environment<br>Malaspina, Andrea; Barts and The London School of Medicine and Dentistry, CPCPH<br>Gallo, Valentina; Barts and The London School of Medicine and Dentistry, CPCPH
Keywords:	Motor neurone disease < NEUROLOGY, SOCIAL MEDICINE, EPIDEMIOLOGY

SCHOLARONE™
Manuscripts

Is charity support distributed equally or fairly? The case of Motor Neurone Disease (MND) Association and its work in Greater London

Anna Gkiouleka[1,2], Allison Manning[3], Dianna Smith[4], Andrea Malaspina[5], Valentina Gallo[1,6,7]

1. Centre for Primary Care and Public Health, Queen Mary University of London, London, UK
2. University of York, York, UK
3. Motor Neurone Disease Association, UK
4. University of Southampton, Southampton, UK
5. Department of Neuroscience and Trauma, Blizard Institute, Queen Mary University of London, London, UK
6. London School of Hygiene and Tropical Medicine, London, UK
7. School of Public Health, Imperial College London, London, UK

Corresponding author:

Dr Valentina Gallo
Centre for Primary Care and Public Health
58, Turner Street
E1 2AB London (UK)
v.gallo@qmul.ac.uk
Tel: +44/(0)20/7882 6135

Word count: 2,843

For peer review only

For peer review only - http://bmjopen.bmj.com/site/about/guidelines.xhtml

Abstract

Objective - There is an immense socio-economic burden for both the patients with Motor Neurone Disease (MND), and their families. The aim of this study is to evaluate the extent to which the provision offered by the MND Association is distributed among MND patients living in the ethnically and socially diverse area of Greater London, according to the patients' socio-economic situation and needs.

Setting – Greater London, where age and sex-adjusted prevalence rates of MND in 2016 were calculated.

Participants – Prevalent MND cases in Greater London, using anonymised data extracted from the Association's database

Exposure – Demographic and socio-economic characteristics

Primary and secondary outcome measures – Receiving an MND Association grant, and the amount of money received

Results - 396 individuals with ALS were detected, the age- and sex-specific prevalence of MND was 4.02 per 100,000 inhabitants; higher among men (5.13 per 100,000) than women (3.01 per 100,000). Men were statistically significantly 40% less likely to receive a grant compared to women; among grant recipients, the younger the age of the participant, the higher the size of the grant received. The Index of multiple Deprivation (IMD) was not associated with either receiving a grant nor the amount of money received, among recipients.

Conclusion - Financial support by the MND Association is provided across individuals and across boroughs regardless of their socio-economic circumstances. Difference that benefits women and younger patients were detected.

Keywords

Motor neurone disease, MND Association, health inequities, Prevalence, London, charity

For peer review only - http://bmjopen.bmj.com/site/about/guidelines.xhtml

Strengths and limitations

- Based on data available to the MND Association, the age- and sex-adjusted prevalence of MND in Greater London was calculated
- The analysis of distribution of financial support by a charity (MND Association) is highly innovative, and no data were previously available
- The overall results of this study inform health care workers about whom is likely to have less access to resources in a urban and diverse context
- The analysis of socio-economic status might be hampered by the use of the index of multiple deprivation at area level as proxy

For peer review only

For peer review only - http://bmjopen.bmj.com/site/about/guidelines.xhtml

Summary box

What is already known on this subject?

Although the socio-economic implications of Motor Neurone Disease (MND) may be similar for all the patients, their impact on the everyday life depends on their socio-economic status. People belonging to the lower socio-economic strata tend to suffer worse health and to have less access to healthcare and health promoting resources. A lower socio-economic status and health disadvantage implies that they have less means to maintain a human quality of life after the disease onset but also that the socio-economic consequences of the disease have a devastating impact on them and their close environment. Ethnicity, age, and sex also contribute shaping inequality in health. Charities, such as MND Association, whose mission is to improve the care and the support for people with MND, their families and carers, and to campaign and raise awareness about the needs of people living with MND, play a key role in addressing such inequalities

What does this study add?

The analysis of financial support offered by the MND Association to people living with MND in greater London showed that women were more likely than men to receive a grant, and – among grant recipients – younger people receive more money than older ones. The finding that people from other/unknown ethnic groups are less likely than whites to receive a grant is likely due to the fact that records of people receiving grants are more likely to be accurate. Importantly, no significant difference was seen among ethnic groups, nor across socio-economic strata

For peer review only

Introduction

The Motor Neurone Disease Association (MND Association) is a charity focused on Motor Neurone Disease (MND) care, research and campaigning with approximately 8,000 members in England, Wales and Northern Ireland. Its mission is to improve the care and the support for people with MND, their families and carers, to campaign and raise awareness about the needs of people living with MND, and to fund and promote relevant research.[1] The current study is part of a broader research project initiated by the Association investigating the socio-demographic characteristics of MND patients in Greater London, the extent of their met and unmet needs, and their utilisation of services and resources offered by the Association.

Amyotrophic lateral sclerosis (ALS), the most common form of Motor Neurone Diseases (MND), is an idiopathic, fatal neurodegenerative disease.[2] Currently, it is estimated that there are approximately 5,000 patients diagnosed with ALS in the UK, with an average age of onset of 55 years. The prevalence of ALS is 4-6 cases per 100,000 population, with a lifetime ALS risk ranging from 1/600 to 1/1000.[1,3,4] Although diverse in its presentation, course and progression, ALS causes progressive muscle atrophy, which results in physical disabilities, loss of independence and eventually death due to respiratory muscle failure. Hence, the socio-economic burden of the disease is immense both for the patients and their carers.[4]

Although the socio-economic implications of ALS may be similar for all the patients (e.g. income reduction, increased health expenses), their impact on the everyday reality of the patients and their families is subject to their socio-economic status prior to the onset of the disease. Current evidence suggests that people belonging to the lower socio-economic strata tend to suffer worse health and to have less access to healthcare and health promoting resources.[5,6] In Britain, health inequalities persist as low income and low levels of subjective financial well-being are associated with poorer health in mid-life and older age.[7] From this perspective, it is likely that MND patients with lower socio-economic status bear an increased health but also socioeconomic disadvantage. This implies that they have less means to maintain a human quality of life after the ALS onset but also that the socio-economic consequences of the disease have a devastating impact on them and their close environment.

For peer review only - http://bmjopen.bmj.com/site/about/guidelines.xhtml

Moreover, studies on ethnic inequalities have revealed considerable gaps in self-rated health comparing ethnic minorities to the white British population. Analysis of 2011 census data has revealed that the White Gypsy or Irish Traveler men and women suffer a significant health disadvantage compared to the White British group, while the rest of the ethnic minorities report similar or better health outcomes than the White British group. This pattern appears stronger among men, as women in most cases either report worse health than the white British group (e.g. Pakistani, Bangladeshi, Arab, Black Caribbean) or their health advantage is smaller than that of their male counterparts (e.g. Black African, Indian).[5,7] At older ages, ethnic minorities suffer higher levels of morbidity compared to the white British population.[5,8] In London, the most ethnically diverse area of England and the region where the highest socio-economic inequalities are observed, ethnic health inequalities are much more severe than in the rest of the country.[8,9] These findings suggest that beyond the pure economic, other dimensions of social positioning like ethnicity, gender or domicile have also a crucial impact on people's health and hence shape health inequalities among social groups. These inequalities translate both into an increased vulnerability to poor health, and into less capabilities to deal with disease.[10] People living with MND in London experience a unique situation of being part of a population structure diverse in terms of demographic characteristics; with a number of socio-economic and health inequalities within groups.

The aim of this project was to evaluate the extent to which the provision offered by the MND Association is distributed among MND patients living in the ethnically and socially diverse area of Greater London, according to the patients' socio-economic situation and needs. Therefore, the main objectives were firstly to estimate the prevalence of MND in London, starting from the data held by the MND Association; and secondly, to examine whether the patients' socio-economic position is associated with becoming a grant recipient, and with the amount of financial support received from the MND Association either in form of cash or equipment (e.g. riser recliners).

Methods

Source if data

For peer review only - http://bmjopen.bmj.com/site/about/guidelines.xhtml

The MND Association database of people living with MND was accessed once the data had been anonymised. Data added to the dataset before 2013 were partially incomplete; therefore, records added on 31st of December 2012 or before and with a date of death before 1st of January 2016 or missing were excluded from the analysis. A cross-sectional analysis to assess period prevalence of MND in London (January to December 2016) was computed including all people who were diagnosed with MND and alive for all or part of the year 2016. MND Association expenditure for grant or equipment in the area of greater London was collected through the Association's database. Information on gender, current age at 30th June 2016, ethnicity, and - where available - disease duration, were collected, in addition to information on financial grants and medical equipment supplied by the Association. Each member was georeferenced at Lower Super Output Area (LSOA 2015) [11] using their postcode. Each LSOA was used to approximate a neighbourhood that has a mean population of 1500.

Participant age was measured in years, disease duration was measured in months from the date of the diagnosis until 31st December 2016 or date of death, if antecedent. Individual level socio-economic status was approximated using the 2015 Index of Multiple Deprivation (IMD) [12] at LSOA level and ethnicity was classified using the categories White, Black, Asian, Mixed ethnicity, and Other/unknown, according to the classification used in 2011 Census. More detailed classification was avoided due to small numbers. The IMD ranks every small area in England from 1 (most deprived area) to 32,844 (least deprived area); this ranking is then divided in deciles which corresponds to most deprived 10 per cent, 20 per cent, etc. Financial support from the MND Association was measured as the total amount of money offered per patient either in form of cash or medical equipment during the one-year period. For the linear regression analysis, this value has been log transformed in order to meet the assumption of normality of distribution.

For the direct standardisation, age- and gender-specific London population by borough was extracted from the census data available from the NOMIS, official labour market statistics [13].

This research has been approved by the Queen Mary, University of London Ethical Committee (QMERC2016/40). No additional data is available for this analysis.

For peer review only - http://bmjopen.bmj.com/site/about/guidelines.xhtml

Patient and Public Involvement

People living with MND were not directly involved in this analysis; however, MND Association commissioned this research to use the evidence on the distribution of awards to inform future policies.

Data Analysis

Individuals with missing information on age (N=71) were recoded with the sex- and borough-specific median age category. A direct standardisation against the WHO standard population separated for men and women was carried out in order to calculate age- and sex- adjusted prevalence rates.

A logistic regression to explore the socio-economic determinants associated with receiving versus non-receiving an MND Association grant was conducted. Among people receiving some form of grants, a linear regression exploring the association of the same determinants and the amount of money awarded to recipients was subsequently run.

A descriptive geographical analysis was conducted using ArcGIS 10.4. Data on age-standardised prevalence of MND was mapped at borough level, due to small patient numbers. Additional mapping of the funding from grants or equipment expenditure per patient was completed at the same scale to identify geographic variation in provision. This data were mapped against the median borough level of the IMD. Statistical analysis was conducted using the STATA 12.1 package.

Data Sharing Statement

Technical appendix, statistical code, and dataset available upon request

Results

A total of 983 observations were extracted from the MND Association dataset, after removal of 4 duplicates and 1 observation with missing value for all dates. A total of 569 were removed because added on or before 31st December 2012 and with missing date of death. Further, 18 records were deleted as they were added on the dataset on or after 1st of January 2017 and no date of diagnosis was recorded. The final analysis was conducted

For peer review only - http://bmjopen.bmj.com/site/about/guidelines.xhtml

on a total of 396 prevalent MND cases (233 men and 163 women) in greater London in 2016.

Direct standardisation was conducted on the total sample of 396 individuals in order to calculate the age- and sex-specific prevalence, by borough. In London, globally, the age-standardised prevalence of MND was 4.02 per 100,000 inhabitants; this was higher among men (5.13 per 100,000) than women (3.01 per 100,000). Standardized sex- and borough specific prevalence per 100,000 is shown in Figure 1. In Table 1 the population of London, number of people living with MND, and their mean and median age by gender is reported alongside the directly standardized prevalence rates per 100,000 inhabitants by borough.

Demographic characteristics of people living with MND in London, are reported in Table 2. Men were more prevalent than women (58.8%), and the overall mean age was 65.7 years (SD 12.7). Whites are the largest ethnic group (32.1%) followed by Asians (6.3%) and Blacks (3.3%). The mean decile of the IMD is 5.4 (SD 2.7), close to the median (Table 2).

The distribution of grant awarding was found to vary across ethnic groups with higher proportions of black (38.5%) and Asian (48.0%) people receiving a grant compared to whites (32.3%) (Table 2). However, as shown by boxplots in Figure 2, among white patients there was the greatest variability in amounts of financial support received with a few cases having received much larger amounts than the average.

In Figure 3, the proportion of people living with MND who have received a grant for the MND Association, by borough, is plotted against the borough specific IMD quantiles. At least one in four people received economic support in four out of six least deprived boroughs, and in only two out of six most deprived boroughs.

Results of the logistic regression investigating factors associated with MND Association grant award are shown in Table 3. Only belonging to an “other” or unknown ethnic group was inversely associated with receiving a grant in the crude analysis. In the adjusted model, men were statistically significantly 40% less likely to receive a grant compared to women; people of other/unknown ethnic background were also 60% less likely to receive a grant compared to whites. When investigating determinants of the amount of money granted, among grant recipients only, age was the only factor found to be negatively significantly associated with amount of grant received: the younger the age of the

For peer review only - http://bmjopen.bmj.com/site/about/guidelines.xhtml

participant, the higher the size of the grant received (Table 3). Interestingly, IMD decile, used as a proxy for socio-economic status of the participants, was not associated with either receiving a grant nor the amount of money received, among recipients.

Discussion

The analysis of financial support offered by the MND Association to people living with MND in greater London showed that women were more likely than men to receive a grant, and – among grant recipients – younger people receive more money than older ones. The finding that people from other/unknown ethnic groups are less likely than whites to receive a grant is likely due to the fact that records of people receiving grants are more likely to be accurate. Importantly, no significant difference was seen among ethnic groups, nor across socio-economic strata.

Gender inequalities

Women's greater probability to receive a grant compared to men could be explained on the basis of culturally dominant constructions of masculinity and femininity that affect men's and women's help seeking behaviours.[14] From this perspective, men, in order to be consistent with the dominant masculine representation, tend to be less eager to seek for medical or social support as such behaviour is associated with weakness and vulnerability, attributes that are traditionally associated with women.[15] In this light, our findings might suggest that men, due to their greater reluctance to seek help, are also less willing to seek for financial support provided by the MND Association. Moreover, due to these gender stereotypes, women in general are also responsible for providing care to the members of their family. This might mean that they are more familiar with healthcare and social support institutions and hence are better informed and skilled in navigating the services. Therefore, the greater probability to receive financial support from the MND association might derive from the fact that women are more informed about the support opportunities or they simply know better where to look for such opportunities compared to men.

On the other hand, another possible interpretation of this association is the presence of a greater need among women compared to men. Given that women are in general more likely to be unemployed, to have more and longer gaps in their working history (e.g. due to pregnancy) or to work part-time and in less regulated sectors than men, implies that they

For peer review only - http://bmjopen.bmj.com/site/about/guidelines.xhtml

also have less access to social provision.[16] So, a greater utilisation of services provided from non-state institutions (i.e. those provided by charities such as the MND Association), might shed light on a gap in social provision that women seek to cover via alternative sources. Future research could reveal which of these explanations apply to MND patients or whether they all apply at the same time. In any case, the findings suggest that the utilisation of the MND Association's financial services is subject to gender differences and this should be taken into consideration in the future communication strategy of the Association.

Age inequalities

The fact that older patients receive smaller amounts of grants from the MND Association might be due to the broader social marginalisation of older people. Older people tend to be less able to maintain social relationships within their communities and this makes them less able to participate in daily activities.[17] They often reside in spatially segregated areas where public and private institutions are also absent.[18] Such circumstances may inhibit older MND patients from seeking and receiving assistance from the MND Association - among other providers-, especially when they also lack strong bonds with family or friends.

Social and ethnic inequalities

The framework of social determinants of health,[19] suggests that socio-economic status is not only an indicator of the actual material and social resources that one has but also of the ability a person has to make use of these resources to achieve their health potential.[20]. In this frame, we would expect that the likelihood of patients to receive financial support from the MND Association as well as the amount of their grant would be subject to their own socio-economic status as an indicator of their everyday circumstances as well as of their capacity to deal with the challenges imposed by the disease. People in lower social strata not only have greater socio-economic needs but have also less capacity to make the ultimate use of the available resources[10]. Hence, in pursuing a fair provision of financial support, we would expect the MND Association to offer grants more often and more generously to patients with a lower socio-economic status. However, finding no evidence for socio-economic and ethnic inequalities in receiving financial support from the MND Association indicates a strategy of 'equal' treatment for all the patients from the side of the Association regardless of the patients' circumstances. Future research with more accurate data in terms of ethnicity and an individual measure of socio-economic status would be

For peer review only - http://bmjopen.bmj.com/site/about/guidelines.xhtml

useful in order to verify this pattern but also to reveal whether this 'equal treatment' approach is the most effective one for the specific population. Equal distribution of resources is not enough for dealing with people who stand in unequal social positions. This might be an issue for consideration for the Association's strategy for providing not only equal but also fair service.

Limitations

Considering the methods used for this analysis, and specifically the exclusion of observations with no recorded date of death, it is also possible that this apparent lack of association is at least partially due to selection bias. If people from lower socio-economic backgrounds and ethnic minorities are more likely to fail to remain engaged with the Association, they are also more likely to have missing data and therefore being underrepresented in the prevalence exercise. This would imply that prevalence among these subgroup would be artificially inflated. The lack of association between IMD and awards may be due to the scale of the analysis and the required assumption that patients in this study had the same socio-economic status as the local population, as IMD is assigned by LSOA (mean population of 1,500). This assumption is potentially an ecological fallacy, as we cannot be certain of individual social status from the data provided [21].

Conclusions

The present results show that provision of financial support by the MND Association is provided across individuals and across boroughs regardless of their socio-economic circumstances. However, it seems that there is a difference across gender and age that benefits women and younger patients, respectively. Ethnic differences have not been observed. Future research with a larger number of cases, spread across multiple regions, and more accurate data on the patients' socio-economic position and ethnic background would allow us to confirm these findings and draw more certain conclusions about the effectiveness of the MND Association's strategy in distributing financial support among patients fairly.

For peer review only - http://bmjopen.bmj.com/site/about/guidelines.xhtml

Acknowledgements

This research has been made possible thanks to a grant of the Motor Neurone Disease (MND) Association to Dr Valentina Gallo (grant number GPPG1G5R).

The funders had no role in influencing data analysis or result interpretation.

Dr Gallo had full access to all of the data in the study and takes responsibility for the integrity of the data and the accuracy of the data analysis. She declares that this manuscript is honest, accurate, and transparent account of the study being reported; that no important aspects of the study have been omitted. All co-authors had full access to the data (including statistical reports and tables), and can take responsibility for the integrity of the data and the accuracy of the data analysis.

Contributorship statement:

Study concept and design: V Gallo

Analysis and interpretation of data: V Gallo, A Giouleka, D Smith

Drafting of the manuscript: A Giuoleka

Data collection: A Manning

Critical revision of the manuscript for important intellectual content: V Gallo, A Malaspina, A Giuoleka, A Manning, D Smith

Conflict of interest statement: we declare that we have no conflict of interests. The financial support from the MND Association has not influenced the submitted work.

For peer review only

Bibliography

1. MNDAssociation. Motor Neurone Disease Association. https://www.mndassociation.org/2017).
2. van Es MA, Hardiman O, Chio A, et al. Amyotrophic lateral sclerosis. *Lancet* 2017.
3. Kiernan MC, Vucic S, Cheah BC, et al. Amyotrophic lateral sclerosis. *Lancet* 2011; **377**(9769): 942-55.
4. Chio A, Logroscino G, Traynor BJ, et al. Global epidemiology of amyotrophic lateral sclerosis: a systematic review of the published literature. *Neuroepidemiology* 2013; **41**(2): 118-30.
5. Watt G. The inverse care law today. *Lancet* 2002; **360**(9328): 252-4.
6. Wilkinson R, Marmot M. Social Determinants of Health - The solid facts. Denmark World Health Organisation 2003.
7. Arber S, Fenn K, Meadows R. Subjective financial well-being, income and health inequalities in mid and later life in Britain. *Soc Sci Med* 2014; **100**: 12-20.
8. Becares L. Which Ethnic groups have the poorest health? Ethnic identity and inequalities in Britain: the dynamics of diversity. Dynamics of diversity: evidence from the 2011 census University of manchester Joseph Rowntree Foundation 2015.
9. Nazroo JY, Williams DR, Marmot M, Wilkinson RG. The social determination of ethnic/racial inequalities in health Social Determinants of Health 2nd ed. Oxford Oxford University Press; 2006.
10. Sen A. Developments as freedom. Oxford Oxford paperbacks 2001.
11. Autority GL. Datastore LSOA Atlas - Greater London authority
12. Gill B. The English Indeces of Deprivation 2015: Departments for Communities and Local Government, 2015.
13. NOMIS. NOMIS - Official Labour Market Statistics.
14. O'Brien R, Hunt K, Hart G. 'It's caveman stuff, but that is to a certain extent how guys still operate': men's accounts of masculinity and help seeking. *Soc Sci Med* 2005; **61**(3): 503-16.
15. Noone JH, Stephens C. Men, masculine identities, and health care utilisation. *Sociol Health Illn* 2008; **30**(5): 711-25.
16. Sainsbury D. Gender, equality and welfare states. Cambridge: Cambridge University Press; 1996.
17. Scharf T. Multiple exclusion and quality of life amongst excluded older people in disadvantages neighborhoods. In: UK OotDPM-, editor. London; 2005.
18. Scharf T, Phillipson C, Smith AE. Social exclusion of older people in deprived urban communities of England. *Eur J Ageing* 2005; **2**(2): 76-87.
19. Dahlgren G, Whitehead M. Rainbow model of health. In: Europe WROf, ed. European Health Policy Conference: Opportunities for the Future Vol 11 –
Intersectoral Action for Health; 1991.
20. Link BG, Phelan J. Social conditions as fundamental causes of disease. *J Health Soc Behav* 1995; **Spec No**: 80-94.
21. Sui D. Ecological fallacy. In: Thrift N, ed. International Encyclopedia of Human Geography. Oxford: Elsevier; 2009: 291-3.

For peer review only - http://bmjopen.bmj.com/site/about/guidelines.xhtml

Table 1: Total population, number of people living with MND, mean, median age and standardised prevalence per 100,000 by gender and borough in greater London in 2016

			Men		Women	
**Borough**	**Total population**	**People with MND**	**Mean/median age**	**Standardised prevalence per 100,000**	**Mean/median age**	**Standardised prevalence per 100,000**
Barking and Dagenham	185,911	12	64/61	6.06	66/67	4.90
Barnet	356,386	22	64/66	7.04	77/74	2.16
Bexley	231,997	14	70/69	5.76	63/63	2.14
Brent	311,215	<10	43/43	1.21	67/67	3.06
Bromley	309,392	23	6365	2.33	70/68	4.89
Camden	220,338	13	62/65	3.41	52/60	4.07
Croydon	363,378	22	72/68	5.48	70/71	3.52
Ealing	338,449	15	53/52	6.08	75/72	1.69
Enfield	312,466	18	63/66	4.11	63/63	4.18
Greenwich	254,557	13	63/66	4.30	63/63	3.28
Hackney	246,270	<10	63/63	2.17	64/65	6.80
Hammersmith and Fulham	182,493	<10	68/68	3.71	60/60	3.25
Haringey	254,926	<10	59/59	1.74	40/40	0.65
Harrow	239,056	15	66/66	7.14	76/76	1.78
Havering	237,232	15	71/72	4.76	69/69	2.29
Hillingdon	273,936	14	6971	5.03	57/54	2.93
Hounslow	253,957	<10	62/62	2.80	50/43	2.80
Islington	206,125	<10	78/78	1.97		0.00
Kensington and Chelsea	158,649	<10	70/67	2.29	87/86	2.38
Kingston upon Thames	160,060	11	61/62	6.93	68/69	3.59
Lambeth	303,086	11	61/60	5.23	63/62	3.10
Lewisham	275,885	12	57/54	5.54	65/60	2.36
Merton	199,693	<10	67/73	4.77	63/51	2.06
Newham	307,984	11	60/60	7.93	63/63	0.82
Redbridge	278,970	24	67/66	7.93	74/79	4.93
Richmond upon Thames	186,990	11	72/74	3.13	71/72	4.36
Southwark	288,283	<10	65/70	4.13	79/79	1.18
Sutton	190,146	12	62/63	6.27	68/70	2.87
Tower Hamlets	254,096	<10	61/59	7.36	48/48	1.15

For peer review only

For peer review only - http://bmjopen.bmj.com/site/about/guidelines.xhtml

Waltham Forest	258,249	11	67/70	4.14	71/72	3.00
Wandsworth	306,995	13	72/73	4.36	69/74	3.80
Westminster	219,396	14	60/60	4.92	66/67	5.95
**Total**	**8,166,566**	**396**	**64 (SD 12)/ 65 (IQR 57-72)**	**5.13**	**68 (SD 13) 70 (IQR 61-76)**	**3.01**

SD=standard deviation; IQR = interquartile range

For peer review only

Table 2: General characteristics of the population living with MND in greater London, and divided by being recipient of a grant from MND Association

Characteristics	All N=396	Grant recipient N=96	Not grant recipient N=300	p-value*
Men (%)	233 (58.8)	49 (51.0)	184 (61.3)	0.075
Age, mean (SD)	65.7 (12.7)	65.0 (13.0)	66.0 (12.6)	0.523
Ethnicity				
White (%)	127 (32.1)	41 (32.3)	86 (67.7)	<0.001
Asian (%)	25 (6.3)	12 (48.0)	13 (52.0)	
Black (%)	13 (3.3)	5 (38.5)	8 (61.5)	
Mixed	7 (1.8)	2 (28.6)	5 (71.4)	
Other/unknown	224 (56.6)	188 (83.9)	36 (16.1)	
Index of Multiple Deprivation				
Decile, mean (SD)	5.4 (2.7)	5.5 (2.7)	5.4 (2.6)	0.773

* p-value from chi-square test for categorical variables, and t-test for continuous variables

For peer review only - http://bmjopen.bmj.com/site/about/guidelines.xhtml

Table 3: OR and relative 95% C.I> coming from logistic regression models investigating determinants of being recipient of an MND grant among prevalent cases of MND in greater London. Beta coefficients and relative 95% C.I. from linear regression models investigating factors associated with the amount of money awarded (in GBP, log transformed)

	**All prevalent cases N=396**		**Grant recipients only N=96**	
	**Logistic regression Crude OR (95% C.I)**	**Logistic regression Adjusted OR (95% C.I)**	**Linear regression Crude β coefficient (95% C.I)**	**Linear regression Adjusted β coefficient (95% C.I)**
Gender (male)	0.66 (0.41-1.04)	**0.60 (0.37-0.98)**	-0.078 (-0.58/0.42)	-0.11 (-0.62/0.39)
Age (years)	0.99 (0.98-1.01)	0.99 (0.98-1.01)	**-0.020 (-0.04/-0.001)**	**-0.02 (-0.04/-0.002)**
Ethnic group				
White	Ref.	Ref.	Ref.	Ref.
Black	1.31 (0.40-4.26)	1.32 (0.39-4.48)	0.52 (-0.65/1.69)	0.33 (-0.86/1.53)
Asian	1.94 (0.81-4.61)	2.18 (0.89-5.32)	-0.08 (-0.89/0.73)	-0.26 (-1.09/0.57)
Mixed	0.84 (0.16-4.51)	0.82 (0.15-4.49)	-0.09 (-1.88/1.69)	-0.30 (-2.09/1.49)
Other/unknown	**0.40 (0.24-0.67)**	**0.40 (0.24-0.68)**	0.17 (-0.39/0.73)	0.10 (-0.46/0.67)
IMD (deciles)	1.01 (0.93-1.10)	1.04 (0.95-1.14)	-0.005 (-0.097/0.087)	0.02 (-0.07/0.12)

For peer review only

Figure legends

Figure 1: Map of greater London showing the prevalence of MND by borough and gender

Figure 2: Boxplots representing total spent in GBP by the MND Association by ethnic group on Greater London (N=396)

Figure 3: Map of greater London showing prportion of people living with MND who received a grant from the MND Association, by borough, over the Index of Multiple Deprivation quintile

For peer review only

For peer review only - http://bmjopen.bmj.com/site/about/guidelines.xhtml

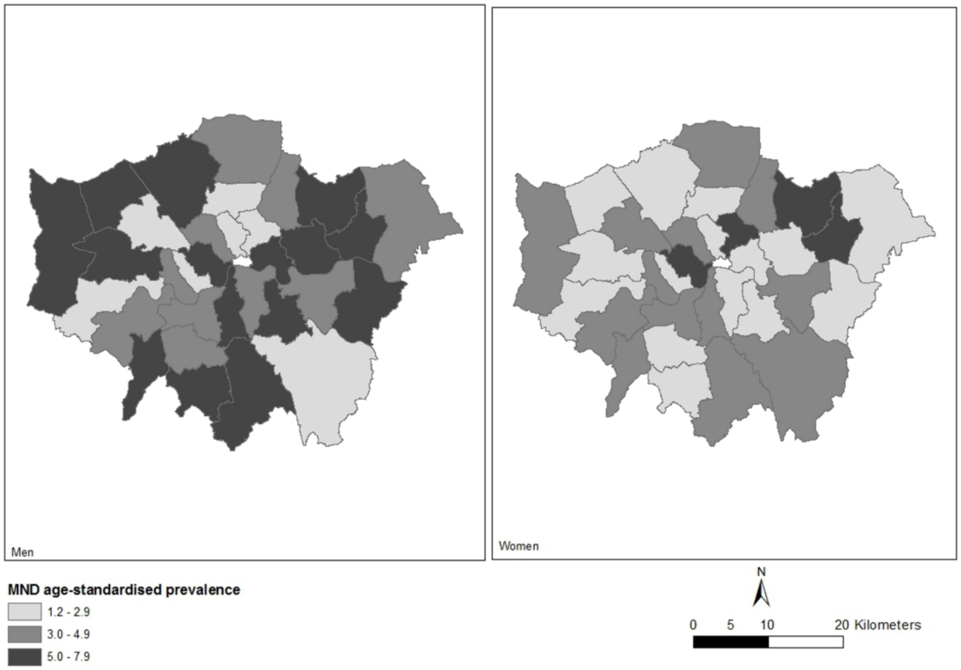

Figure 1: Map of greater London showing the prevalence of MND by borough and gender

205x145mm (300 x 300 DPI)

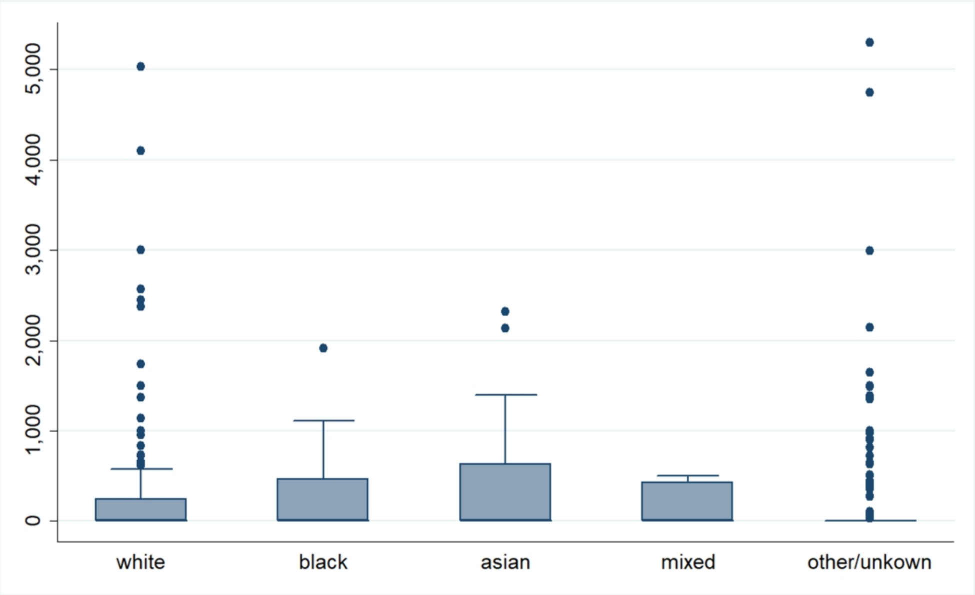

Figure 2: Boxplots representing total spent in GBP by the MND Association by ethnic group on Greater London (N=396)

205x131mm (300 x 300 DPI)

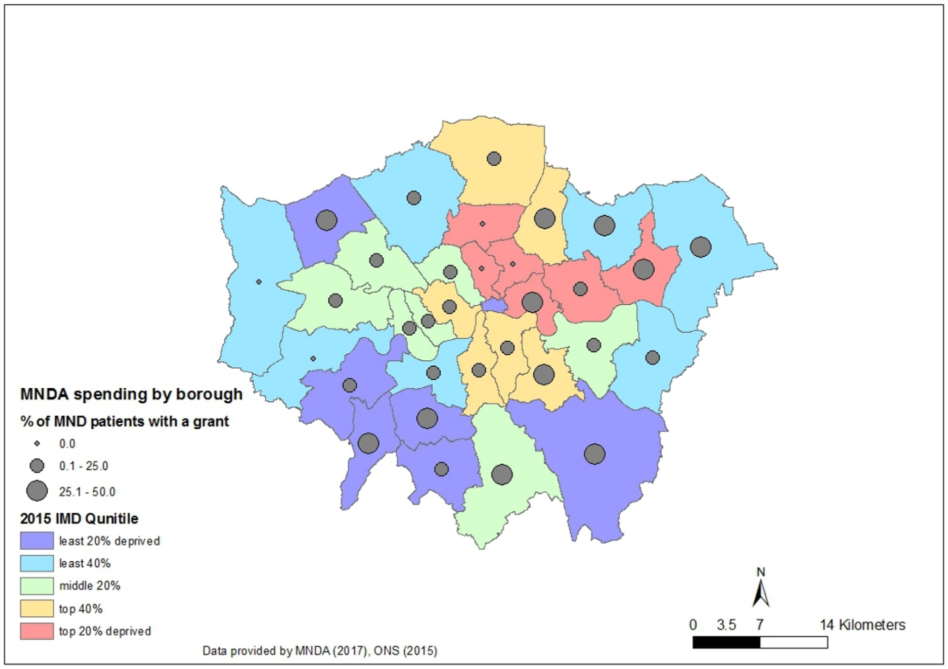

Figure 3: Map of greater London showing prportion of people living with MND who received a grant from the MND Association, by borough, over the Index of Multiple Deprivation quintile

207x146mm (300 x 300 DPI)

STROBE Statement—Checklist of items that should be included in reports of ***cross-sectional studies***

	Item No	Recommendation	Manuscript
**Title and abstract**	1	(*a*) Indicate the study's design with a commonly used term in the title or the abstract	N/A
		(*b*) Provide in the abstract an informative and balanced summary of what was done and what was found	p. 2
**Introduction**			
Background/rationale	2	Explain the scientific background and rationale for the investigation being reported	p. 5-6
Objectives	3	State specific objectives, including any prespecified hypotheses	p. 6
**Methods**			
Study design	4	Present key elements of study design early in the paper	p. 7
Setting	5	Describe the setting, locations, and relevant dates, including periods of recruitment, exposure, follow-up, and data collection	p. 6-7
Participants	6	(*a*) Give the eligibility criteria, and the sources and methods of selection of participants	p. 7
Variables	7	Clearly define all outcomes, exposures, predictors, potential confounders, and effect modifiers. Give diagnostic criteria, if applicable	p. 7
Data sources/ measurement	8*	For each variable of interest, give sources of data and details of methods of assessment (measurement). Describe comparability of assessment methods if there is more than one group	p. 7
Bias	9	Describe any efforts to address potential sources of bias	p. 7
Study size	10	Explain how the study size was arrived at	p. 6-7
Quantitative variables	11	Explain how quantitative variables were handled in the analyses. If applicable, describe which groupings were chosen and why	p. 7
Statistical methods	12	(*a*) Describe all statistical methods, including those used to control for confounding	p. 8
		(*b*) Describe any methods used to examine subgroups and interactions	N/A
		(*c*) Explain how missing data were addressed	p. 7
		(*d*) If applicable, describe analytical methods taking account of sampling strategy	N/A
		(*e*) Describe any sensitivity analyses	N/A
**Results**			
Participants	13*	(a) Report numbers of individuals at each stage of study—eg numbers potentially eligible, examined for eligibility, confirmed eligible, included in the study, completing follow-up, and analysed	p. 8
		(b) Give reasons for non-participation at each stage	N/A
		(c) Consider use of a flow diagram	N/A
Descriptive data	14*	(a) Give characteristics of study participants (eg demographic, clinical, social) and information on exposures and potential confounders	p. 8
		(b) Indicate number of participants with missing data for each variable of interest	p. 8

For peer review only - http://bmjopen.bmj.com/site/about/guidelines.xhtml

Outcome data	15*	Report numbers of outcome events or summary measures	p. 8
Main results	16	(*a*) Give unadjusted estimates and, if applicable, confounder-adjusted estimates and their precision (eg, 95% confidence interval). Make clear which confounders were adjusted for and why they were included	Table 2
		(*b*) Report category boundaries when continuous variables were categorized	N/A
		(*c*) If relevant, consider translating estimates of relative risk into absolute risk for a meaningful time period	N/A
Other analyses	17	Report other analyses done—eg analyses of subgroups and interactions, and sensitivity analyses	N/A
**Discussion**			
Key results	18	Summarise key results with reference to study objectives	p. 9-10
Limitations	19	Discuss limitations of the study, taking into account sources of potential bias or imprecision. Discuss both direction and magnitude of any potential bias	p. 12
Interpretation	20	Give a cautious overall interpretation of results considering objectives, limitations, multiplicity of analyses, results from similar studies, and other relevant evidence	p. 12
Generalisability	21	Discuss the generalisability (external validity) of the study results	p. 10-11
**Other information**			
Funding	22	Give the source of funding and the role of the funders for the present study and, if applicable, for the original study on which the present article is based	p. 13

*Give information separately for exposed and unexposed groups.

Note: An Explanation and Elaboration article discusses each checklist item and gives methodological background and published examples of transparent reporting. The STROBE checklist is best used in conjunction with this article (freely available on the Web sites of PLoS Medicine at http://www.plosmedicine.org/, Annals of Internal Medicine at http://www.annals.org/, and Epidemiology at http://www.epidem.com/). Information on the STROBE Initiative is available at www.strobe-statement.org.

For peer review only

For peer review only - http://bmjopen.bmj.com/site/about/guidelines.xhtml

Charity financial support to Motor Neurone Disease (MND) patients in Greater London: the impact of patients' socio-economic status – a cross-sectional study

Journal:	*BMJ Open*
Manuscript ID	bmjopen-2018-022462.R1
Article Type:	Research
Date Submitted by the Author:	10-Jul-2018
Complete List of Authors:	Giouleka, Anna; Barts and The London School of Medicine and Dentistry, CPCPH<br>Manning, Alison; Motor Neurone Disease Association<br>Smith, Dianna; University of Southampton, Geography & Environment<br>Malaspina, Andrea; Barts and The London School of Medicine and Dentistry, CPCPH<br>Gallo, Valentina; Barts and The London School of Medicine and Dentistry, CPCPH
**Primary Subject Heading**:	Epidemiology
Secondary Subject Heading:	Epidemiology, Health services research, Neurology, Sociology, Public health
Keywords:	Motor neurone disease < NEUROLOGY, SOCIAL MEDICINE, EPIDEMIOLOGY

Charity financial support to Motor Neurone Disease (MND) patients in Greater London: the impact of patients' socio-economic status – a cross-sectional study

Anna Gkiouleka[1,2], Allison Manning[3], Dianna Smith[4], Andrea Malaspina[5], Valentina Gallo[1,6,7]

1. Centre for Primary Care and Public Health, Queen Mary University of London, London, UK
2. University of York, York, UK
3. Motor Neurone Disease Association, UK
4. University of Southampton, Southampton, UK
5. Department of Neuroscience and Trauma, Blizard Institute, Queen Mary University of London, London, UK
6. London School of Hygiene and Tropical Medicine, London, UK
7. School of Public Health, Imperial College London, London, UK

Corresponding author:

Dr Valentina Gallo
Centre for Primary Care and Public Health
58, Turner Street
E1 2AB London (UK)
v.gallo@qmul.ac.uk
Tel: +44/(0)20/7882 6135

Word count: 2,843

For peer review only

For peer review only - http://bmjopen.bmj.com/site/about/guidelines.xhtml

Abstract

Objective - There is an immense socio-economic burden for both the patients with Motor Neurone Disease (MND), and their families. The aim of this study is to evaluate the extent to which the provision offered by the MND Association is distributed among MND patients living in the ethnically and socially diverse area of Greater London, according to the patients' socio-economic situation and needs.

Setting – Greater London, where age and sex-adjusted prevalence rates of MND in 2016 were calculated.

Participants – Prevalent MND cases in Greater London, using anonymised data extracted from the Association's database

Exposure – Demographic and socio-economic characteristics

Primary and secondary outcome measures – Receiving an MND Association grant, and the amount of money received

Results - 396 individuals with ALS were detected, the age- and sex-specific prevalence of MND was 4.02 per 100,000 inhabitants; higher among men (5.13 per 100,000) than women (3.01 per 100,000). Men were statistically significantly 40% less likely to receive a grant compared to women; among grant recipients, the younger the age of the participant, the higher the size of the grant received. The Index of multiple Deprivation (IMD) was not associated with either receiving a grant nor the amount of money received, among recipients.

Conclusion - Financial support by the MND Association is provided across individuals and across boroughs regardless of their socio-economic circumstances. Difference that benefits women and younger patients were detected.

Keywords

Motor neurone disease, MND Association, health inequities, Prevalence, London, charity

For peer review only

Strengths and limitations

- Based on data available to the MND Association, the age- and sex-adjusted prevalence of MND in Greater London could be calculated
- Data available in the MND Association database cover the entirety of greater London allowing for comparison across boroughs and highly socio-economically diverse areas
- The analysis of socio-economic status might be limited by the use of the index of multiple deprivation at area level as proxy
- Information bias (more accurate information were collected for people who received a grant compared to those who did not) and selection bias (lack of information about people living with MND in greater London, but not MND Association members) cannot be entirely ruled out

For peer review only

For peer review only - http://bmjopen.bmj.com/site/about/guidelines.xhtml

Summary box

What is already known on this subject?

Although the socio-economic implications of Motor Neurone Disease (MND) may be similar for all the patients, their impact on the everyday life depends on their socio-economic status. People belonging to the lower socio-economic strata tend to suffer worse health and to have less access to healthcare and health promoting resources. A lower socio-economic status and health disadvantage implies that they have less means to maintain a human quality of life after the disease onset but also that the socio-economic consequences of the disease have a devastating impact on them and their close environment. Ethnicity, age, and sex also contribute shaping inequality in health. Charities, such as the MND Association, whose mission is to improve the care and the support for people with MND, their families and carers, and to campaign and raise awareness about the needs of people living with MND, play a key role in addressing such inequalities

What does this study add?

The analysis of financial support offered by the MND Association to people living with MND in greater London showed that women were more likely than men to receive a grant, and – among grant recipients – younger people receive more money than older ones. The finding that people from other/unknown ethnic groups are less likely than whites to receive a grant is likely due to the fact that records of people receiving grants are more likely to be accurate. Importantly, no significant difference was seen among ethnic groups, nor across socio-economic strata

For peer review only

Introduction

The Motor Neurone Disease Association (MND Association) is a charity focused on Motor Neurone Disease (MND) care, research and campaigning with approximately 8,000 members in England, Wales and Northern Ireland. Its mission is to improve the care and the support for people with MND, their families and carers, to campaign and raise awareness about the needs of people living with MND, and to fund and promote relevant research.[1] The current study is part of a broader research project initiated by the Association investigating the socio-demographic characteristics of MND patients in Greater London, the extent of their met and unmet needs, and their utilisation of services and resources offered by the Association.

Amyotrophic lateral sclerosis (ALS), the most common form of Motor Neurone Diseases, is an idiopathic, fatal neurodegenerative disease.[2] Currently, it is estimated that there are approximately 5,000 patients diagnosed with ALS in the UK, with an average age of onset of 55 years. The prevalence of ALS is 4-6 cases per 100,000 population, with a lifetime ALS risk ranging from 1/600 to 1/1000.[1,3,4] Although diverse in its presentation, course and progression, ALS causes progressive muscle atrophy, which results in physical disabilities, loss of independence and eventually death due to respiratory muscle failure. Hence, the socio-economic burden of the disease is immense both for the patients and their carers.[4]

Although the socio-economic implications of ALS may be similar for all the patients (e.g. income reduction, increased health expenses), their impact on the everyday reality of the patients and their families is subject to their socio-economic status prior to the onset of the disease. Current evidence suggests that people belonging to the lower socio-economic strata tend to suffer worse health and to have less access to healthcare and health promoting resources.[5,6] In Britain, health inequalities persist as low income and low levels of subjective financial well-being are associated with poorer health in mid-life and older age.[7] From this perspective, it is likely that MND patients with lower socio-economic status bear an increased health but also socioeconomic disadvantage. This implies that they have less means to maintain a human quality of life after the ALS onset but also that the socio-economic consequences of the disease have a devastating impact on them and their close environment.

Moreover, studies on ethnic inequalities have revealed considerable gaps in self-rated health comparing ethnic minorities to the white British population. Analysis of 2011 census

For peer review only - http://bmjopen.bmj.com/site/about/guidelines.xhtml

data has revealed that the White Gypsy or Irish Traveler men and women suffer a significant health disadvantage compared to the White British group, while the rest of the ethnic minorities report similar or better health outcomes than the White British group. This pattern appears stronger among men, as women in most cases either report worse health than the white British group (e.g. Pakistani, Bangladeshi, Arab, Black Caribbean) or their health advantage is smaller than that of their male counterparts (e.g. Black African, Indian).[5,7] At older ages, ethnic minorities suffer higher levels of morbidity compared to the white British population.[5,8] In London, the most ethnically diverse area of England and the region where the highest socio-economic inequalities are observed, ethnic health inequalities are much more severe than in the rest of the country.[8,9] These findings suggest that beyond the pure economic, other dimensions of social positioning like ethnicity, gender or domicile have also a crucial impact on people's health and hence shape health inequalities among social groups. These inequalities translate both into an increased vulnerability to poor health, and into less capabilities to deal with disease.[10] People living with MND in London experience a unique situation of being part of a population structure diverse in terms of demographic characteristics; with a number of socio-economic and health inequalities within groups.

The MND Association acknowledging both the financial implications of the disease for the patients' lives and the existing socio-economic inequalities in Greater London concentrates its efforts in providing direct (i.e. grants) or indirect (i.e. equipment) financial support to MND patients in the area. For this, all patients are informed by their care centre about the existence of the Association and those patients who express their interest in initiating a contact get referred to it by the care centre. Next, the Association sends to them extensive information material about the challenges of the disease, the available services offered by the NHS and those offered by the MND Association including financial support. Thus, patients can claim for it according to their needs. Their claims are usually made directly to the regional care development adviser in a personal manner and they get approved up to a maximum of 2,000 pounds. The MND Association is the only large association working for people living with MND in London.

The overarching aim of this study was to evaluate if support given by the MND Association was distributed equally or fairly among people living with MND in London. As a consequence we aimed to describe the provision offered by the MND Association among MND patients living in the ethnically and socially diverse area of Greater London, according to the patients' demographic and socio-economic situation and needs. The main

For peer review only - http://bmjopen.bmj.com/site/about/guidelines.xhtml

objectives were firstly to estimate the prevalence of MND in London, starting from the data held by the MND Association; and secondly, to explore whether the patients' socio-economic position, gender, and age were associated with the likelihood of receiving a grant, and with the amount of the grant received from the MND Association either in form of cash or equipment (e.g. riser recliners).

Methods

Source of data

The MND Association database of people living with MND was accessed once the data had been anonymised. Data added to the dataset before 2013 were partially incomplete; therefore, records added on 31st of December 2012 or before and with a date of death before 1st of January 2016 or missing were excluded from the analysis. Period prevalence of MND in London (January to December 2016) was computed including all people who were diagnosed with MND and alive for all or part of the year 2016. MND Association expenditure for grant or equipment in the area of greater London was collected through the Association's database that was updated with accurate information about financial grants and medical equipment supplied by the Association's staff for routing expenditure monitoring and then extracted for the purposes of this study. Information on gender was complete, while information on current age at 30th June 2016 was available for all but 71 patients. On the other hand, ethnicity and disease duration was mainly available for the grant receivers, implying a more consistent communication between the patients and the Association. Each patient was georeferenced at Lower Super Output Area (LSOA 2015) [11] using their postcode. Each LSOA was used to approximate a neighbourhood that has a mean population of 1500.

Participant age was measured in years, disease duration was measured in months from the date of the diagnosis until 31st December 2016 or date of death, if antecedent. Individual level socio-economic status was approximated using the 2015 Index of Multiple Deprivation (IMD) [12] at LSOA level and ethnicity was classified using the categories White, Black, Asian, Mixed ethnicity, and Other/unknown, according to the classification used in 2011 Census. More detailed classification was avoided due to small numbers. The IMD ranks every small area in England from 1 (most deprived area) to 32,844 (least deprived area); this ranking is then divided in deciles which corresponds to most deprived 10 per

For peer review only - http://bmjopen.bmj.com/site/about/guidelines.xhtml

cent, 20 per cent, etc. Financial support from the MND Association was measured as the total amount of money offered per patient either in form of cash or medical equipment during the one-year period. For the linear regression analysis, this value has been log transformed in order to meet the assumption of normality of distribution.

For the direct standardisation, age- and gender-specific London population by borough was extracted from the census data available from the NOMIS, official labour market statistics [13].

This research has been approved by the Queen Mary, University of London Ethical Committee (QMERC2016/40). No additional data is available for this analysis.

Data Analysis

Individuals with missing information on age (N=71) were recoded with the sex- and borough-specific median age category. A direct standardisation against the WHO standard population separated for men and women was carried out in order to calculate age- and sex- adjusted prevalence rates.

A logistic regression to explore the socio-economic determinants associated with receiving versus non-receiving an MND Association grant was conducted. Among people receiving some form of grants, a linear regression exploring the association of the same determinants and the amount of money awarded to recipients was subsequently run.

Non-linearity of the association between grant amount and age has been tested adding the age-squared term. In addition, a quintile analysis of the grant amount has been conducted to assess if any specific group is more likely to receive exceptionally large amount of money.

A descriptive geographical analysis was conducted using ArcGIS 10.4. Data on age-standardised prevalence of MND was mapped at borough level, due to small patient numbers. Additional mapping of the funding from grants or equipment expenditure per patient was completed at the same scale to identify geographic variation in provision. This data were mapped against the median borough level of the IMD. Statistical analysis was conducted using the STATA 12.1 package.

For peer review only - http://bmjopen.bmj.com/site/about/guidelines.xhtml

Results

A total of 983 observations were extracted from the MND Association dataset, after removal of 4 duplicates and 1 observation with missing value for all dates. A total of 569 were removed because added on or before 31st December 2012 and with missing date of death. Further, 18 records were deleted as they were added on the dataset on or after 1st of January 2017 and no date of diagnosis was recorded. The final analysis was conducted on a total of 396 prevalent MND cases (233 men and 163 women) in greater London in 2016.

Direct standardisation was conducted on the total sample of 396 individuals in order to calculate the age- and sex-specific prevalence, by borough. In London, globally, the age-standardised prevalence of MND was 4.02 per 100,000 inhabitants; this was higher among men (5.13 per 100,000) than women (3.01 per 100,000). Standardized sex- and borough specific prevalence per 100,000 is shown in Figure 1. In Table 1 the population of London, number of people living with MND, and their mean and median age by gender is reported alongside the directly standardized prevalence rates per 100,000 inhabitants by borough.

Demographic characteristics of people living with MND in London, are reported in Table 2. Men were more prevalent than women (58.8%), and the overall mean age was 65.7 years (SD 12.7). Whites are the largest ethnic group (32.1%) followed by Asians (6.3%) and Blacks (3.3%). The mean decile of the IMD is 5.4 (SD 2.7), close to the median (Table 2).

The distribution of grant awarding was found to vary across ethnic groups with higher proportions of black (38.5%) and Asian (48.0%) people receiving a grant compared to whites (32.3%) (Table 2). However, as shown by boxplots in Figure 2, among white patients there was the greatest variability in amounts of financial support received with a few cases having received much larger amounts than the average.

In Figure 3, the proportion of people living with MND who have received a grant for the MND Association, by borough, is plotted against the borough specific IMD quantiles. At least one in four people received economic support in four out of six least deprived boroughs, and in only two out of six most deprived boroughs.

Results of the logistic regression investigating factors associated with MND Association grant award are shown in Table 3. Only belonging to an “other” or unknown ethnic group

For peer review only - http://bmjopen.bmj.com/site/about/guidelines.xhtml

was inversely associated with receiving a grant in the crude analysis. In the adjusted model, men were statistically significantly 40% less likely to receive a grant compared to women; people of other/unknown ethnic background were also 60% less likely to receive a grant compared to whites. When investigating determinants of the amount of money granted, among grant recipients only, age was the only factor found to be negatively significantly associated with amount of grant received: the younger the age of the participant, the higher the size of the grant received (Table 3). Adding age as a quadratic term did not show a better fit compared to the linear association; a quintile analysis for IMD yield to very similar results (results now shown). Interestingly, IMD decile, used as a proxy for socio-economic status of the participants, was not associated with either receiving a grant nor the amount of money received, among recipients.

Discussion

The analysis of financial support offered by the MND Association to people living with MND in greater London showed that women were more likely than men to receive a grant, and – among grant recipients – younger people receive more money than older ones. The finding that people from other/unknown ethnic groups are less likely than whites to receive a grant is likely due an information bias, in fact, records of people receiving grants are more likely to be accurate. Importantly, no significant difference was seen among ethnic groups, nor across socio-economic strata. However, these findings need to be interpreted with caution as no information was available on people living with MND in London who were not members of MND Association. The potential selection bias underlying this could have biased towards the null a possible positive association between receiving a grant and socio-economic position.

Gender inequalities

Women's greater probability to receive a grant compared to men could be explained on the basis of culturally dominant constructions of masculinity and femininity that affect men's and women's help seeking behaviours.[14] From this perspective, men, in order to be consistent with the dominant masculine representation, tend to be less eager to seek for medical or social support as such behaviour is associated with weakness and vulnerability, attributes that are traditionally associated with women.[15] In this light, our findings might suggest that men, due to their greater reluctance to seek help, are also less willing to seek for financial support provided by the MND Association. Moreover, due to these gender

For peer review only - http://bmjopen.bmj.com/site/about/guidelines.xhtml

stereotypes, women in general are also responsible for providing care to the members of their family. This might mean that they are more familiar with healthcare and social support institutions and hence are better informed and skilled in navigating the services. Therefore, the greater probability to receive financial support from the MND association might derive from the fact that women are more informed about the support opportunities or they simply know better where to look for such opportunities compared to men.

On the other hand, another possible interpretation of this association is the presence of a greater need among women compared to men. Given that women are in general more likely to be unemployed, to have more and longer gaps in their working history (e.g. due to pregnancy) or to work part-time and in less regulated sectors than men, implies that they also have less access to social provision.[16] So, a greater utilisation of services provided from non-state institutions (i.e. those provided by charities such as the MND Association), might shed light on a gap in social provision that women seek to cover via alternative sources. Future research could reveal which of these explanations apply to MND patients or whether they all apply at the same time. In any case, the findings suggest that the utilisation of the MND Association's financial services is subject to gender differences and this should be taken into consideration in the future communication strategy of the Association.

However, a cautious interpretation of this findings is needed, as we cannot exclude that men are overrepresented in this sample due to a selection bias. This would have biased the observed association which would look stronger than is in reality.

Age inequalities

The fact that older patients receive smaller amounts of grants from the MND Association might be due to the broader social marginalisation of older people. Older people tend to be less able to maintain social relationships within their communities and this makes them less able to participate in daily activities.[17] They often reside in spatially segregated areas where public and private institutions are also absent.[18] Such circumstances may inhibit older MND patients from seeking and receiving assistance from the MND Association - among other providers-, especially when they also lack strong bonds with family or friends. Again, it is not possible to rule out that these findings could at least in part due to selection bias in case older people were more likely to be overrepresented in the observed sample.

For peer review only - http://bmjopen.bmj.com/site/about/guidelines.xhtml

Social and ethnic inequalities

The framework of social determinants of health,[19] suggests that socio-economic status is not only an indicator of the actual material and social resources that one has but also of the ability a person has to make use of these resources to achieve their health potential.[20]. In this frame, we would expect that the likelihood of patients to receive financial support from the MND Association as well as the amount of their grant would be subject to their own socio-economic status as an indicator of their everyday circumstances as well as of their capacity to deal with the challenges imposed by the disease. People in lower social strata not only have greater socio-economic needs but have also less capacity to make the ultimate use of the available resources[10]. Hence, in pursuing a fair provision of financial support, we would expect the MND Association to offer grants more often and more generously to patients with a lower socio-economic status. However, finding no evidence for socio-economic and ethnic inequalities in receiving financial support from the MND Association indicates a strategy of 'equal' treatment for all the patients from the side of the Association regardless of the patients' circumstances. However, our estimates could be biased towards the null, if we assume the presence of a selection bias making more wealthy people more likely to access the MND Association (and therefore being included in the present study) compared to more deprived people. Future research with more accurate data in terms of ethnicity and an individual measure of socio-economic status would be useful in order to verify this pattern but also to reveal whether this 'equal treatment' approach is the most effective one for the specific population. Equal distribution of resources is not enough for dealing with people who stand in unequal social positions. This might be an issue for consideration for the Association's strategy for providing not only equal but also fair service. Also, a population-based registry would reduce the risk of selection bias and allow more precise estimations.

Limitations

Considering the methods used for this analysis, and specifically the exclusion of observations with no recorded date of death, it is possible that this apparent lack of association is at least partially due to selection bias. If people from lower socio-economic backgrounds and ethnic minorities are more likely to fail to remain engaged with the Association, they are also more likely to have missing data and therefore being underrepresented in the prevalence exercise. This would imply that prevalence among these subgroup would be artificially inflated. Moreover, it is difficult to estimate

For peer review only - http://bmjopen.bmj.com/site/about/guidelines.xhtml

generalisability of these results, as data on prevalent MND cases not members of MND Association are lacking. This could be further source of selection bias as those people are more likely to be less educated or migrants. The lack of association between IMD and awards may be due to the scale of the analysis and the required assumption that patients in this study had the same socio-economic status as the local population, as IMD is assigned by LSOA (mean population of 1,500). This assumption is potentially subject to ecological fallacy [21], , as individual socio-economic status cannot be always estimated accurately from IMD [22] Moreover, the limited sample size of the analysis among grant recipients does not allow detection of smaller effects, which could be only achieved by increasing the power. Finally, although all the patients who get referred to the MND Association receive the information material about the available financial support, assuming that all of them read the material and are equally able to make contact with the Association in order to claim financial support is unlikely. So, future research should also explore the extent to which the information about the services available by the Association is distributed equally and whether there are certain patients who are less likely to be aware of their entitlements.

Conclusions

The present results show that provision of financial support by the MND Association is provided across individuals and across boroughs regardless of their socio-economic circumstances. However, it seems that there is a difference across gender and age that benefits women and younger patients, respectively. Ethnic differences have not been observed. Future research with a larger number of cases, spread across multiple regions, and more accurate data on the patients' socio-economic position and ethnic background would allow us to confirm these findings and draw more certain conclusions about the effectiveness of the MND Association's strategy in distributing financial support among patients fairly.

For peer review only - http://bmjopen.bmj.com/site/about/guidelines.xhtml

Acknowledgements

This research has been made possible thanks to a grant of the Motor Neurone Disease (MND) Association to Dr Valentina Gallo (grant number GPPG1G5R).

The funders had no role in influencing data analysis or result interpretation.

Dr Gallo had full access to all of the data in the study and takes responsibility for the integrity of the data and the accuracy of the data analysis. She declares that this manuscript is honest, accurate, and transparent account of the study being reported; that no important aspects of the study have been omitted. All co-authors had full access to the data (including statistical reports and tables), and can take responsibility for the integrity of the data and the accuracy of the data analysis.

Contributorship statement:

Study concept and design: V Gallo

Analysis and interpretation of data: V Gallo, A Giouleka, D Smith

Drafting of the manuscript: A Giuoleka

Data collection: A Manning

Critical revision of the manuscript for important intellectual content: A Malaspina, A Manning, D Smith, V Gallo

Conflict of interest statement:

We declare that we have no conflict of interests. The financial support from the MND Association has not influenced the submitted work.

Funding:

This article is part of the project "Social inequalities in accessing services provided by the MND Association" funded by the MND Association (GPPG1G5R)

Data sharing:

No additional unpublished data is available for this study.

For peer review only

Bibliography

1. MND Association available at https://www.mndassociation.org/2017).
2. van Es MA, Hardiman O, Chio A, et al. Amyotrophic lateral sclerosis. *Lancet* 2017.
3. Kiernan MC, Vucic S, Cheah BC, et al. Amyotrophic lateral sclerosis. *Lancet* 2011; **377**(9769): 942-55.
4. Chio A, Logroscino G, Traynor BJ, et al. Global epidemiology of amyotrophic lateral sclerosis: a systematic review of the published literature. *Neuroepidemiology* 2013; **41**(2): 118-30.
5. Watt G. The inverse care law today. *Lancet* 2002; **360**(9328): 252-4.
6. Wilkinson R, Marmot M. Social Determinants of Health - The solid facts. Denmark World Health Organisation 2003.
7. Arber S, Fenn K, Meadows R. Subjective financial well-being, income and health inequalities in mid and later life in Britain. *Soc Sci Med* 2014; **100**: 12-20.
8. Becares L. Which Ethnic groups have the poorest health? Ethnic identity and inequalities in Britain: the dynamics of diversity. Dynamics of diversity: evidence from the 2011 census University of manchester Joseph Rowntree Foundation 2015.
9. Nazroo JY, Williams DR, Marmot M, Wilkinson RG. The social determination of ethnic/racial inequalities in health Social Determinants of Health 2nd ed. Oxford Oxford University Press; 2006.
10. Sen A. Developments as freedom. Oxford Oxford paperbacks 2001.
11. Autority GL. Datastore LSOA Atlas - Greater London authority
12. Gill B. The English Indeces of Deprivation 2015: Departments for Communities and Local Government, 2015.
13. NOMIS. NOMIS - Official Labour Market Statistics.
14. O'Brien R, Hunt K, Hart G. 'It's caveman stuff, but that is to a certain extent how guys still operate': men's accounts of masculinity and help seeking. *Soc Sci Med* 2005; **61**(3): 503-16.
15. Noone JH, Stephens C. Men, masculine identities, and health care utilisation. *Sociol Health Illn* 2008; **30**(5): 711-25.
16. Sainsbury D. Gender, equality and welfare states. Cambridge: Cambridge University Press; 1996.
17. Scharf T. Multiple exclusion and quality of life amongst excluded older people in disadvantages neighborhoods. In: UK OotDPM-, editor. London; 2005.
18. Scharf T, Phillipson C, Smith AE. Social exclusion of older people in deprived urban communities of England. *Eur J Ageing* 2005; **2**(2): 76-87.
19. Dahlgren G, Whitehead M. Rainbow model of health. In: Europe WROf, ed. European Health Policy Conference: Opportunities for the Future Vol 11 –
Intersectoral Action for Health; 1991.
20. Link BG, Phelan J. Social conditions as fundamental causes of disease. *J Health Soc Behav* 1995; **Spec No**: 80-94.
21. Sui D. Ecological fallacy. In: Thrift N, ed. International Encyclopedia of Human Geography. Oxford: Elsevier; 2009: 291-3.
22. Deas, I., B. Robson, C. Wong and M. Bradford (2003). "Measuring neighbourhood deprivation: a critique of the Index of Multiple Deprivation." Environment and PlanningC: Government and Policy **21**: 883-903.

For peer review only - http://bmjopen.bmj.com/site/about/guidelines.xhtml

Table 1: Total population, number of people living with MND, mean, median age and standardised prevalence per 100,000 by gender and borough in greater London in 2016

			Men		Women	
**Borough**	**Total population**	**People with MND**	**Mean/median age**	**Standardised prevalence per 100,000**	**Mean/median age**	**Standardised prevalence per 100,000**
Barking and Dagenham	185,911	12	64/61	6.06	66/67	4.90
Barnet	356,386	22	64/66	7.04	77/74	2.16
Bexley	231,997	14	70/69	5.76	63/63	2.14
Brent	311,215	<10	43/43	1.21	67/67	3.06
Bromley	309,392	23	6365	2.33	70/68	4.89
Camden	220,338	13	62/65	3.41	52/60	4.07
Croydon	363,378	22	72/68	5.48	70/71	3.52
Ealing	338,449	15	53/52	6.08	75/72	1.69
Enfield	312,466	18	63/66	4.11	63/63	4.18
Greenwich	254,557	13	63/66	4.30	63/63	3.28
Hackney	246,270	<10	63/63	2.17	64/65	6.80
Hammersmith and Fulham	182,493	<10	68/68	3.71	60/60	3.25
Haringey	254,926	<10	59/59	1.74	40/40	0.65
Harrow	239,056	15	66/66	7.14	76/76	1.78
Havering	237,232	15	71/72	4.76	69/69	2.29
Hillingdon	273,936	14	6971	5.03	57/54	2.93
Hounslow	253,957	<10	62/62	2.80	50/43	2.80
Islington	206,125	<10	78/78	1.97		0.00
Kensington and Chelsea	158,649	<10	70/67	2.29	87/86	2.38
Kingston upon Thames	160,060	11	61/62	6.93	68/69	3.59
Lambeth	303,086	11	61/60	5.23	63/62	3.10
Lewisham	275,885	12	57/54	5.54	65/60	2.36
Merton	199,693	<10	67/73	4.77	63/51	2.06
Newham	307,984	11	60/60	7.93	63/63	0.82
Redbridge	278,970	24	67/66	7.93	74/79	4.93
Richmond upon Thames	186,990	11	72/74	3.13	71/72	4.36
Southwark	288,283	<10	65/70	4.13	79/79	1.18
Sutton	190,146	12	62/63	6.27	68/70	2.87
Tower Hamlets	254,096	<10	61/59	7.36	48/48	1.15
Waltham Forest	258,249	11	67/70	4.14	71/72	3.00

For peer review only

Wandsworth	306,995	13	72/73	4.36	69/74	3.80
Westminster	219,396	14	60/60	4.92	66/67	5.95
**Total**	**8,166,566**	**396**	**64 (SD 12)/ 65 (IQR 57-72)**	**5.13**	**68 (SD 13) 70 (IQR 61-76)**	**3.01**

SD=standard deviation; IQR = interquartile range

For peer review only

Table 2: General characteristics of the population living with MND in greater London, and divided by being recipient of a grant from MND Association

Characteristics	All N=396	Grant recipient N=96	Not grant recipient N=300	p-value*
Men (%)	233 (58.8)	49 (51.0)	184 (61.3)	0.075
Age, mean (SD)	65.7 (12.7)	65.0 (13.0)	66.0 (12.6)	0.523
Ethnicity				
White (%)	127 (32.1)	41 (32.3)	86 (67.7)	<0.001
Asian (%)	25 (6.3)	12 (48.0)	13 (52.0)	
Black (%)	13 (3.3)	5 (38.5)	8 (61.5)	
Mixed	7 (1.8)	2 (28.6)	5 (71.4)	
Other/unknown	224 (56.6)	188 (83.9)	36 (16.1)	
Index of Multiple Deprivation				
Decile, mean (SD)	5.4 (2.7)	5.5 (2.7)	5.4 (2.6)	0.773

* p-value from chi-square test for categorical variables, and t-test for continuous variables

For peer review only

Table 3: OR and relative 95% C.I> coming from logistic regression models investigating determinants of being recipient of an MND grant among prevalent cases of MND in greater London. Beta coefficients and relative 95% C.I. from linear regression models investigating factors associated with the amount of money awarded (in GBP, log transformed)

	**All prevalent cases N=396**		**Grant recipients only N=96**	
	**Logistic regression Crude OR (95% C.I)**	**Logistic regression Adjusted OR (95% C.I)**	**Linear regression Crude β coefficient (95% C.I)**	**Linear regression Adjusted β coefficient (95% C.I)**
Male gender	0.66 (0.41-1.04)	**0.60 (0.37-0.98)**	-0.078 (-0.58/0.42)	-0.11 (-0.62/0.39)
Age (years)	0.99 (0.98-1.01)	0.99 (0.98-1.01)	**-0.020 (-0.04/-0.001)**	**-0.02 (-0.04/-0.002)**
Ethnic group				
White	Ref.	Ref.	Ref.	Ref.
Black	1.31 (0.40-4.26)	1.32 (0.39-4.48)	0.52 (-0.65/1.69)	0.33 (-0.86/1.53)
Asian	1.94 (0.81-4.61)	2.18 (0.89-5.32)	-0.08 (-0.89/0.73)	-0.26 (-1.09/0.57)
Mixed	0.84 (0.16-4.51)	0.82 (0.15-4.49)	-0.09 (-1.88/1.69)	-0.30 (-2.09/1.49)
Other/unknown	**0.40 (0.24-0.67)**	**0.40 (0.24-0.68)**	0.17 (-0.39/0.73)	0.10 (-0.46/0.67)
IMD (deciles)	1.01 (0.93-1.10)	1.04 (0.95-1.14)	-0.005 (-0.097/0.087)	0.02 (-0.07/0.12)

For peer review only

Figure legends

Figure 1: Map of greater London showing the prevalence of MND by borough and gender

Figure 2: Boxplots representing total spent in GBP by the MND Association by ethnic group on Greater London (N=396)

Figure 3: Map of greater London showing prportion of people living with MND who received a grant from the MND Association, by borough, over the Index of Multiple Deprivation quintile

For peer review only

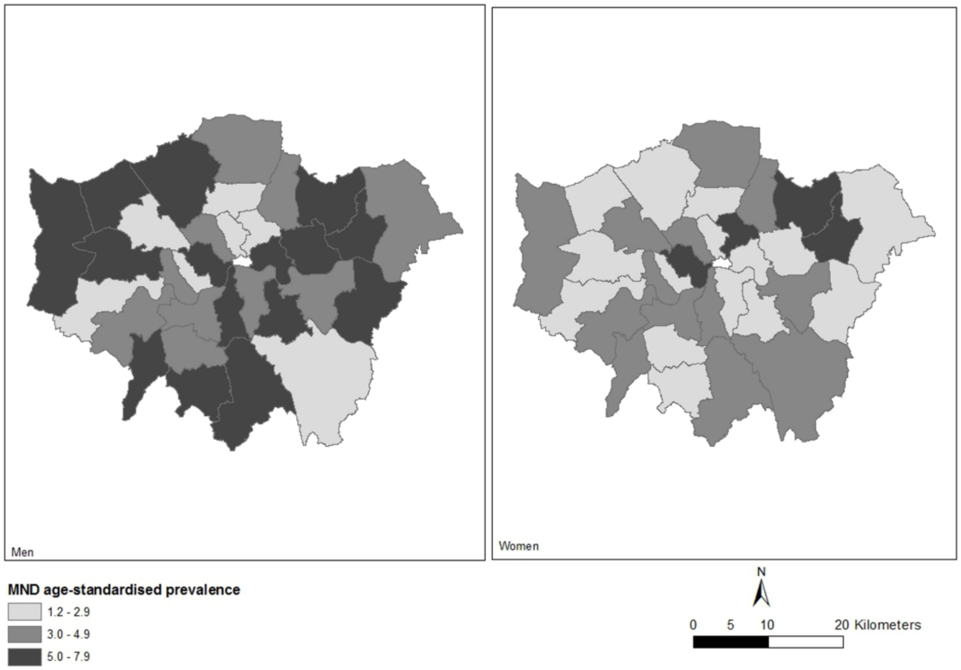

Figure 1: Map of greater London showing the prevalence of MND by borough and gender

205x145mm (300 x 300 DPI)

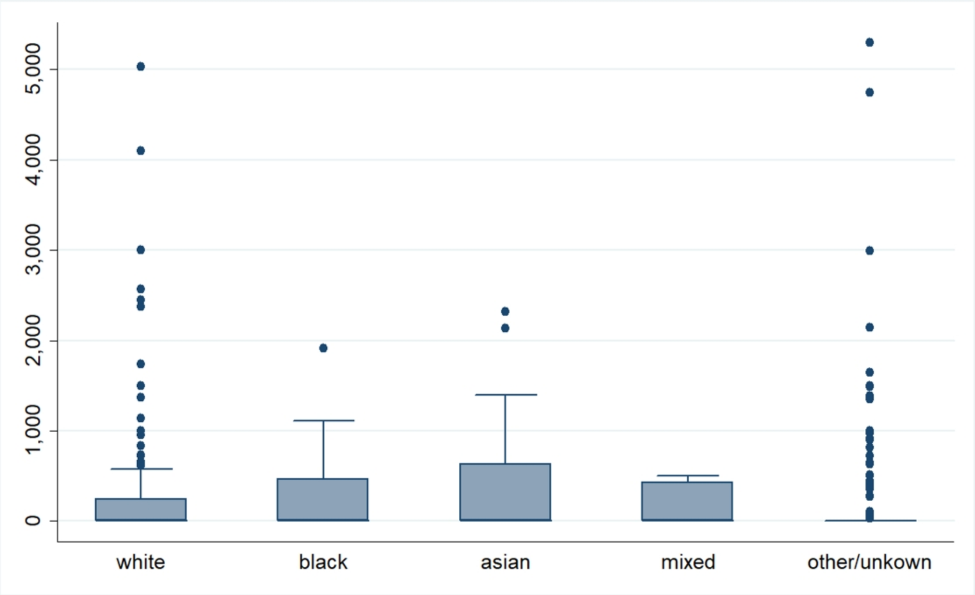

Figure 2: Boxplots representing total spent in GBP by the MND Association by ethnic group on Greater London (N=396)

205x131mm (300 x 300 DPI)

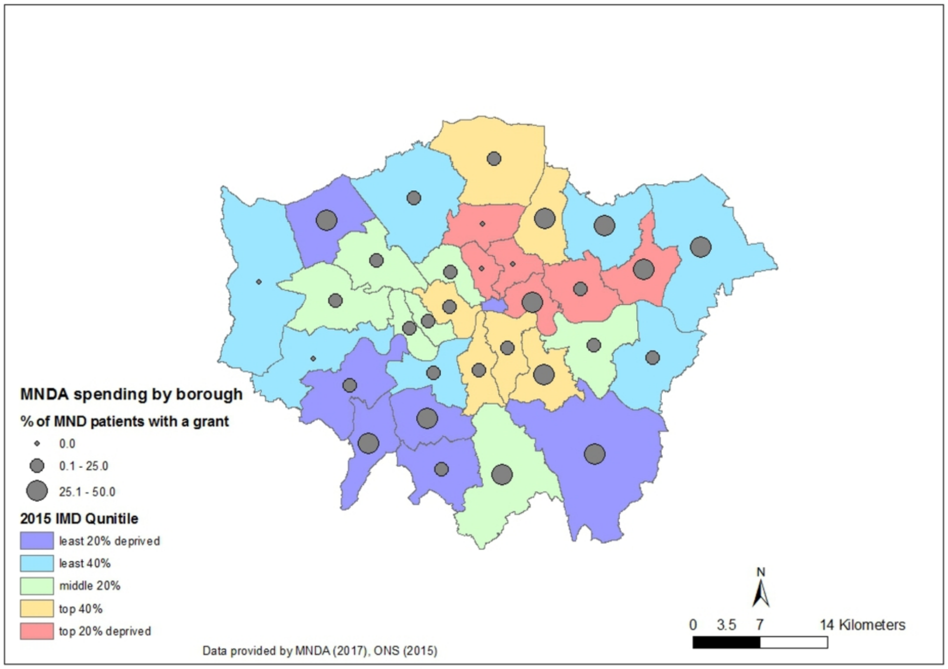

Figure 3: Map of greater London showing prportion of people living with MND who received a grant from the MND Association, by borough, over the Index of Multiple Deprivation quintile

207x146mm (300 x 300 DPI)

STROBE Statement—Checklist of items that should be included in reports of ***cross-sectional studies***

	Item No	Recommendation	Manuscript
**Title and abstract**	1	(*a*) Indicate the study's design with a commonly used term in the title or the abstract	N/A
		(*b*) Provide in the abstract an informative and balanced summary of what was done and what was found	p. 2
**Introduction**			
Background/rationale	2	Explain the scientific background and rationale for the investigation being reported	p. 5-6
Objectives	3	State specific objectives, including any prespecified hypotheses	p. 6
**Methods**			
Study design	4	Present key elements of study design early in the paper	p. 7
Setting	5	Describe the setting, locations, and relevant dates, including periods of recruitment, exposure, follow-up, and data collection	p. 6-7
Participants	6	(*a*) Give the eligibility criteria, and the sources and methods of selection of participants	p. 7
Variables	7	Clearly define all outcomes, exposures, predictors, potential confounders, and effect modifiers. Give diagnostic criteria, if applicable	p. 7
Data sources/ measurement	8*	For each variable of interest, give sources of data and details of methods of assessment (measurement). Describe comparability of assessment methods if there is more than one group	p. 7
Bias	9	Describe any efforts to address potential sources of bias	p. 7
Study size	10	Explain how the study size was arrived at	p. 6-7
Quantitative variables	11	Explain how quantitative variables were handled in the analyses. If applicable, describe which groupings were chosen and why	p. 7
Statistical methods	12	(*a*) Describe all statistical methods, including those used to control for confounding	p. 8
		(*b*) Describe any methods used to examine subgroups and interactions	N/A
		(*c*) Explain how missing data were addressed	p. 7
		(*d*) If applicable, describe analytical methods taking account of sampling strategy	N/A
		(*e*) Describe any sensitivity analyses	N/A
**Results**			
Participants	13*	(a) Report numbers of individuals at each stage of study—eg numbers potentially eligible, examined for eligibility, confirmed eligible, included in the study, completing follow-up, and analysed	p. 8
		(b) Give reasons for non-participation at each stage	N/A
		(c) Consider use of a flow diagram	N/A
Descriptive data	14*	(a) Give characteristics of study participants (eg demographic, clinical, social) and information on exposures and potential confounders	p. 8
		(b) Indicate number of participants with missing data for each variable of interest	p. 8

For peer review only

For peer review only - http://bmjopen.bmj.com/site/about/guidelines.xhtml

Outcome data	15*	Report numbers of outcome events or summary measures	p. 8
Main results	16	(*a*) Give unadjusted estimates and, if applicable, confounder-adjusted estimates and their precision (eg, 95% confidence interval). Make clear which confounders were adjusted for and why they were included	Table 2
		(*b*) Report category boundaries when continuous variables were categorized	N/A
		(*c*) If relevant, consider translating estimates of relative risk into absolute risk for a meaningful time period	N/A
Other analyses	17	Report other analyses done—eg analyses of subgroups and interactions, and sensitivity analyses	N/A
**Discussion**			
Key results	18	Summarise key results with reference to study objectives	p. 9-10
Limitations	19	Discuss limitations of the study, taking into account sources of potential bias or imprecision. Discuss both direction and magnitude of any potential bias	p. 12
Interpretation	20	Give a cautious overall interpretation of results considering objectives, limitations, multiplicity of analyses, results from similar studies, and other relevant evidence	p. 12
Generalisability	21	Discuss the generalisability (external validity) of the study results	p. 10-11
**Other information**			
Funding	22	Give the source of funding and the role of the funders for the present study and, if applicable, for the original study on which the present article is based	p. 13

*Give information separately for exposed and unexposed groups.

Note: An Explanation and Elaboration article discusses each checklist item and gives methodological background and published examples of transparent reporting. The STROBE checklist is best used in conjunction with this article (freely available on the Web sites of PLoS Medicine at http://www.plosmedicine.org/, Annals of Internal Medicine at http://www.annals.org/, and Epidemiology at http://www.epidem.com/). Information on the STROBE Initiative is available at www.strobe-statement.org.

For peer review only

For peer review only - http://bmjopen.bmj.com/site/about/guidelines.xhtml

Charity financial support to Motor Neurone Disease (MND) patients in Greater London: the impact of patients' socio-economic status – a cross-sectional study

Journal:	*BMJ Open*
Manuscript ID	bmjopen-2018-022462.R2
Article Type:	Research
Date Submitted by the Author:	24-Oct-2018
Complete List of Authors:	Gkiouleka, Anna; Barts and The London School of Medicine and Dentistry, CPCPH; University of York<br>Manning, Alison; Motor Neurone Disease Association<br>Smith, Dianna; University of Southampton, Geography & Environment<br>Malaspina, Andrea; Barts and The London School of Medicine and Dentistry, CPCPH<br>Gallo, Valentina; Barts and The London School of Medicine and Dentistry, CPCPH
**Primary Subject Heading**:	Epidemiology
Secondary Subject Heading:	Epidemiology, Health services research, Neurology, Sociology, Public health
Keywords:	Motor neurone disease < NEUROLOGY, SOCIAL MEDICINE, EPIDEMIOLOGY

SCHOLARONE™
Manuscripts

Charity financial support to Motor Neurone Disease (MND) patients in Greater London: the impact of patients' socio-economic status – a cross-sectional study

Anna Gkiouleka[1,2], Alison Manning[3], Dianna Smith[4], Andrea Malaspina[5], Valentina Gallo[1,6,7]

1. Centre for Primary Care and Public Health, Queen Mary University of London, London, UK
2. University of York, York, UK
3. Motor Neurone Disease Association, UK
4. University of Southampton, Southampton, UK
5. Department of Neuroscience and Trauma, Blizard Institute, Queen Mary University of London, London, UK
6. London School of Hygiene and Tropical Medicine, London, UK
7. School of Public Health, Imperial College London, London, UK

Corresponding author:

Dr Valentina Gallo
Centre for Primary Care and Public Health
58, Turner Street
E1 2AB London (UK)
v.gallo@qmul.ac.uk
Tel: +44/(0)20/7882 6135

Word count: 2,843

For peer review only

Abstract

Objective - There is an immense socio-economic burden for both the patients with Motor Neurone Disease (MND), and their families. The aim of this study is to evaluate the extent to which the provision offered by the MND Association is distributed among MND patients living in the ethnically and socially diverse area of Greater London, according to the patients' socio-economic situation and needs.

Setting – Greater London, where age and sex-adjusted prevalence rates of MND in 2016 were calculated.

Participants – Prevalent MND cases in Greater London, using anonymised data extracted from the Association's database

Exposure – Demographic and socio-economic characteristics

Primary and secondary outcome measures – Receiving an MND Association grant, and the amount of money received

Results - 396 individuals with ALS were detected, the age- and sex-specific prevalence of MND was 4.02 per 100,000 inhabitants; higher among men (5.13 per 100,000) than women (3.01 per 100,000). Men were statistically significantly 40% less likely to receive a grant compared to women; among grant recipients, the younger the age of the participant, the higher the size of the grant received. The Index of multiple Deprivation (IMD) was not associated with either receiving a grant nor the amount of money received, among recipients.

Conclusion - Financial support by the MND Association is provided across individuals and across boroughs regardless of their socio-economic circumstances. Difference that benefits women and younger patients were detected.

Keywords

Motor neurone disease, MND Association, health inequities, Prevalence, London, charity

For peer review only

Strengths and limitations

- Based on data available to the MND Association, the age- and sex-adjusted prevalence of MND in Greater London could be calculated
- Data available in the MND Association database cover the entirety of greater London allowing for comparison across boroughs and highly socio-economically diverse areas
- The analysis of socio-economic status might be limited by the use of the index of multiple deprivation at area level as proxy
- Information bias (more accurate information were collected for people who received a grant compared to those who did not) and selection bias (lack of information about people living with MND in greater London, but not MND Association members) cannot be entirely ruled out

For peer review only

Introduction

The Motor Neurone Disease Association (MND Association) is a charity focused on Motor Neurone Disease (MND) care, research and campaigning with approximately 8,000 members in England, Wales and Northern Ireland. Its mission is to improve the care and the support for people with MND, their families and carers, to campaign and raise awareness about the needs of people living with MND, and to fund and promote relevant research.[1] The current study is part of a broader research project initiated by the Association investigating the socio-demographic characteristics of MND patients in Greater London, the extent of their met and unmet needs, and their utilisation of services and resources offered by the Association.

Amyotrophic lateral sclerosis (ALS), the most common form of Motor Neurone Diseases, is an idiopathic, fatal neurodegenerative disease.[2] Currently, it is estimated that there are approximately 5,000 patients diagnosed with ALS in the UK, with an average age of onset of 55 years. The prevalence of ALS is 4-6 cases per 100,000 population, with a lifetime ALS risk ranging from 1/600 to 1/1000.[1,3,4] Although diverse in its presentation, course and progression, ALS causes progressive muscle atrophy, which results in physical disabilities, loss of independence and eventually death due to respiratory muscle failure. Hence, the socio-economic burden of the disease is immense both for the patients and their carers.[4]

Although the socio-economic implications of ALS may be similar for all the patients (e.g. income reduction, increased health expenses), their impact on the everyday reality of the patients and their families is subject to their socio-economic status prior to the onset of the disease. Current evidence suggests that people belonging to the lower socio-economic strata tend to suffer worse health and to have less access to healthcare and health promoting resources.[5,6] In Britain, health inequalities persist as low income and low levels of subjective financial well-being are associated with poorer health in mid-life and older age.[7] From this perspective, it is likely that MND patients with lower socio-economic status bear an increased health but also socioeconomic disadvantage. This implies that they have less means to maintain a human quality of life after the ALS onset

For peer review only

but also that the socio-economic consequences of the disease have a devastating impact on them and their close environment.

Moreover, studies on ethnic inequalities have revealed considerable gaps in self-rated health comparing ethnic minorities to the white British population. Analysis of 2011 census data has revealed that the White Gypsy or Irish Traveler men and women suffer a significant health disadvantage compared to the White British group, while the rest of the ethnic minorities report similar or better health outcomes than the White British group. This pattern appears stronger among men, as women in most cases either report worse health than the white British group (e.g. Pakistani, Bangladeshi, Arab, Black Caribbean) or their health advantage is smaller than that of their male counterparts (e.g. Black African, Indian).[5,7] At older ages, ethnic minorities suffer higher levels of morbidity compared to the white British population.[5,8] In London, the most ethnically diverse area of England and the region where the highest socio-economic inequalities are observed, ethnic health inequalities are much more severe than in the rest of the country.[8,9] These findings suggest that beyond the pure economic, other dimensions of social positioning like ethnicity, gender or domicile have also a crucial impact on people's health and hence shape health inequalities among social groups. These inequalities translate both into an increased vulnerability to poor health, and into less capabilities to deal with disease.[10] People living with MND in London experience a unique situation of being part of a population structure diverse in terms of demographic characteristics; with a number of socio-economic and health inequalities within groups.

The MND Association acknowledging both the financial implications of the disease for the patients' lives and the existing socio-economic inequalities in Greater London concentrates its efforts in providing direct (i.e. grants) or indirect (i.e. equipment) financial support to MND patients in the area. For this, all patients are informed by their care centre about the existence of the Association and those patients who express their interest in initiating a contact get referred to it by the care centre. Next, the Association sends to them extensive information material about the challenges of the disease, the available services offered by the NHS and those offered by the MND Association including financial support. Thus, patients can claim for it according to their needs. Their claims are usually

For peer review only

made directly to the regional care development adviser in a personal manner and they get approved up to a maximum of 2,000 pounds. The MND Association is the only large association working for people living with MND in London.

The overarching aim of this study was to evaluate if support given by the MND Association was distributed equally or fairly among people living with MND in London. As a consequence we aimed to describe the provision offered by the MND Association among MND patients living in the ethnically and socially diverse area of Greater London, according to the patients' demographic and socio-economic situation and needs. The main objectives were firstly to estimate the prevalence of MND in London, starting from the data held by the MND Association; and secondly, to explore whether the patients' socio-economic position, gender, and age were associated with the likelihood of receiving a grant, and with the amount of the grant received from the MND Association either in form of cash or equipment (e.g. riser recliners).

Methods

Source of data

The MND Association database of people living with MND was accessed once the data had been anonymised. Data added to the dataset before 2013 were partially incomplete; therefore, records added on 31st of December 2012 or before and with a date of death before 1st of January 2016 or missing were excluded from the analysis. Period prevalence of MND in London (January to December 2016) was computed including all people who were diagnosed with MND and alive for all or part of the year 2016. MND Association expenditure for grant or equipment in the area of greater London was collected through the Association's database that was updated with accurate information about financial grants and medical equipment supplied by the Association's staff for routing expenditure monitoring and then extracted for the purposes of this study. Information on gender was complete, while information on current age at 30th June 2016 was available for all but 71 patients. On the other hand, ethnicity and disease duration was mainly available for the grant receivers, implying a more consistent communication between the patients and the

For peer review only - http://bmjopen.bmj.com/site/about/guidelines.xhtml

Association. Each patient was georeferenced at Lower Super Output Area (LSOA 2015) [11] using their postcode. Each LSOA was used to approximate a neighbourhood that has a mean population of 1500.

Participant age was measured in years, disease duration was measured in months from the date of the diagnosis until 31st December 2016 or date of death, if antecedent. Individual level socio-economic status was approximated using the 2015 Index of Multiple Deprivation (IMD) [12] at LSOA level and ethnicity was classified using the categories White, Black, Asian, Mixed ethnicity, and Other/unknown, according to the classification used in 2011 Census. More detailed classification was avoided due to small numbers. The IMD ranks every small area in England from 1 (most deprived area) to 32,844 (least deprived area); this ranking is then divided in deciles which corresponds to most deprived 10 per cent, 20 per cent, etc. Financial support from the MND Association was measured as the total amount of money offered per patient either in form of cash or medical equipment during the one-year period. For the linear regression analysis, this value has been log transformed in order to meet the assumption of normality of distribution.

For the direct standardisation, age- and gender-specific London population by borough was extracted from the census data available from the NOMIS, official labour market statistics [13].

This research has been approved by the Queen Mary, University of London Ethical Committee (QMERC2016/40). No additional data is available for this analysis.

Data Analysis

Individuals with missing information on age (N=71) were recoded with the sex- and borough-specific median age category. A direct standardisation against the WHO standard population separated for men and women was carried out in order to calculate age- and sex- adjusted prevalence rates.

For peer review only

A logistic regression to explore the socio-economic determinants associated with receiving versus non-receiving an MND Association grant was conducted. Among people receiving some form of grants, a linear regression exploring the association of the same determinants and the amount of money awarded to recipients was subsequently run.

Non-linearity of the association between grant amount and age has been tested adding the age-squared term. In addition, a quintile analysis of the grant amount has been conducted to assess if any specific group is more likely to receive exceptionally large amount of money.

A descriptive geographical analysis was conducted using ArcGIS 10.4. Data on age-standardised prevalence of MND was mapped at borough level, due to small patient numbers. Additional mapping of the funding from grants or equipment expenditure per patient was completed at the same scale to identify geographic variation in provision. This data were mapped against the median borough level of the IMD. Statistical analysis was conducted using the STATA 12.1 package.

Patient and Public Involvement

There was not patient public involvement in the study design or analysis. Results will be made available to patients through the MND Association website

Results

A total of 983 observations were extracted from the MND Association dataset, after removal of 4 duplicates and 1 observation with missing value for all dates. A total of 569 were removed because added on or before 31st December 2012 and with missing date of death. Further, 18 records were deleted as they were added on the dataset on or after 1st of January 2017 and no date of diagnosis was recorded. The final analysis was conducted on a total of 396 prevalent MND cases (233 men and 163 women) in greater London in 2016.

For peer review only

Direct standardisation was conducted on the total sample of 396 individuals in order to calculate the age- and sex-specific prevalence, by borough. In London, globally, the age-standardised prevalence of MND was 4.02 per 100,000 inhabitants; this was higher among men (5.13 per 100,000) than women (3.01 per 100,000). Standardized sex- and borough specific prevalence per 100,000 is shown in Figure 1. In Table 1 the population of London, number of people living with MND, and their mean and median age by gender is reported alongside the directly standardized prevalence rates per 100,000 inhabitants by borough.

Demographic characteristics of people living with MND in London, are reported in Table 2. Men were more prevalent than women (58.8%), and the overall mean age was 65.7 years (SD 12.7). Whites are the largest ethnic group (32.1%) followed by Asians (6.3%) and Blacks (3.3%). The mean decile of the IMD is 5.4 (SD 2.7), close to the median (Table 2).

The distribution of grant awarding was found to vary across ethnic groups with higher proportions of black (38.5%) and Asian (48.0%) people receiving a grant compared to whites (32.3%) (Table 2). However, as shown by boxplots in Figure 2, among white patients there was the greatest variability in amounts of financial support received with a few cases having received much larger amounts than the average.

In Figure 3, the proportion of people living with MND who have received a grant for the MND Association, by borough, is plotted against the borough specific IMD quantiles. At least one in four people received economic support in four out of six least deprived boroughs, and in only two out of six most deprived boroughs.

Results of the logistic regression investigating factors associated with MND Association grant award are shown in Table 3. Only belonging to an "other" or unknown ethnic group was inversely associated with receiving a grant in the crude analysis. In the adjusted model, men were statistically significantly 40% less likely to receive a grant compared to women; people of other/unknown ethnic background were also 60% less likely to receive a grant compared to whites. When investigating determinants of the amount of money

For peer review only

granted, among grant recipients only, age was the only factor found to be negatively significantly associated with amount of grant received: the younger the age of the participant, the higher the size of the grant received (Table 3). Adding age as a quadratic term did not show a better fit compared to the linear association; a quintile analysis for IMD yield to very similar results (results now shown). Interestingly, IMD decile, used as a proxy for socio-economic status of the participants, was not associated with either receiving a grant nor the amount of money received, among recipients.

Discussion

The analysis of financial support offered by the MND Association to people living with MND in greater London showed that women were more likely than men to receive a grant, and – among grant recipients – younger people receive more money than older ones. The finding that people from other/unknown ethnic groups are less likely than whites to receive a grant is likely due an information bias, in fact, records of people receiving grants are more likely to be accurate. Importantly, no significant difference was seen among ethnic groups, nor across socio-economic strata. However, these findings need to be interpreted with caution as no information was available on people living with MND in London who were not members of MND Association. The potential selection bias underlying this could have biased towards the null a possible positive association between receiving a grant and socio-economic position.

Gender inequalities

Women's greater probability to receive a grant compared to men could be explained on the basis of culturally dominant constructions of masculinity and femininity that affect men's and women's help seeking behaviours.[14] From this perspective, men, in order to be consistent with the dominant masculine representation, tend to be less eager to seek for medical or social support as such behaviour is associated with weakness and vulnerability, attributes that are traditionally associated with women.[15] In this light, our findings might suggest that men, due to their greater reluctance to seek help, are also less willing to seek for financial support provided by the MND Association. Moreover, due

For peer review only

to these gender stereotypes, women in general are also responsible for providing care to the members of their family. This might mean that they are more familiar with healthcare and social support institutions and hence are better informed and skilled in navigating the services. Therefore, the greater probability to receive financial support from the MND association might derive from the fact that women are more informed about the support opportunities or they simply know better where to look for such opportunities compared to men.

On the other hand, another possible interpretation of this association is the presence of a greater need among women compared to men. Given that women are in general more likely to be unemployed, to have more and longer gaps in their working history (e.g. due to pregnancy) or to work part-time and in less regulated sectors than men, implies that they also have less access to social provision.[16] So, a greater utilisation of services provided from non-state institutions (i.e. those provided by charities such as the MND Association), might shed light on a gap in social provision that women seek to cover via alternative sources. Future research could reveal which of these explanations apply to MND patients or whether they all apply at the same time. In any case, the findings suggest that the utilisation of the MND Association's financial services is subject to gender differences and this should be taken into consideration in the future communication strategy of the Association.

However, a cautious interpretation of this findings is needed, as we cannot exclude that men are overrepresented in this sample due to a selection bias. This would have biased the observed association which would look stronger than is in reality.

Age inequalities

The fact that older patients receive smaller amounts of grants from the MND Association might be due to the broader social marginalisation of older people. Older people tend to be less able to maintain social relationships within their communities and this makes them less able to participate in daily activities.[17] They often reside in spatially segregated areas where public and private institutions are also absent.[18] Such circumstances may inhibit older MND patients from seeking and receiving assistance from the MND Association -

For peer review only

among other providers-, especially when they also lack strong bonds with family or friends. Again, it is not possible to rule out that these findings could at least in part due to selection bias in case older people were more likely to be overrepresented in the observed sample.

Social and ethnic inequalities

The framework of social determinants of health,[19] suggests that socio-economic status is not only an indicator of the actual material and social resources that one has but also of the ability a person has to make use of these resources to achieve their health potential.[20]. In this frame, we would expect that the likelihood of patients to receive financial support from the MND Association as well as the amount of their grant would be subject to their own socio-economic status as an indicator of their everyday circumstances as well as of their capacity to deal with the challenges imposed by the disease. People in lower social strata not only have greater socio-economic needs but have also less capacity to make the ultimate use of the available resources[10]. Hence, in pursuing a fair provision of financial support, we would expect the MND Association to offer grants more often and more generously to patients with a lower socio-economic status. However, finding no evidence for socio-economic and ethnic inequalities in receiving financial support from the MND Association indicates a strategy of 'equal' treatment for all the patients from the side of the Association regardless of the patients' circumstances. However, our estimates could be biased towards the null, if we assume the presence of a selection bias making more wealthy people more likely to access the MND Association (and therefore being included in the present study) compared to more deprived people. Future research with more accurate data in terms of ethnicity and an individual measure of socio-economic status would be useful in order to verify this pattern but also to reveal whether this 'equal treatment' approach is the most effective one for the specific population. Equal distribution of resources is not enough for dealing with people who stand in unequal social positions. This might be an issue for consideration for the Association's strategy for providing not only equal but also fair service. Also, a population-based registry would reduce the risk of selection bias and allow more precise estimations.

For peer review only - http://bmjopen.bmj.com/site/about/guidelines.xhtml

Limitations

Considering the methods used for this analysis, and specifically the exclusion of observations with no recorded date of death, it is possible that this apparent lack of association is at least partially due to selection bias. If people from lower socio-economic backgrounds and ethnic minorities are more likely to fail to remain engaged with the Association, they are also more likely to have missing data and therefore being underrepresented in the prevalence exercise. This would imply that prevalence among these subgroup would be artificially inflated. Moreover, it is difficult to estimate generalisability of these results, as data on prevalent MND cases not members of MND Association are lacking. This could be further source of selection bias as those people are more likely to be less educated or migrants. The lack of association between IMD and awards may be due to the scale of the analysis and the required assumption that patients in this study had the same socio-economic status as the local population, as IMD is assigned by LSOA (mean population of 1,500). This assumption is potentially subject to ecological fallacy [21], , as individual socio-economic status cannot be always estimated accurately from IMD [22] Moreover, the limited sample size of the analysis among grant recipients does not allow detection of smaller effects, which could be only achieved by increasing the power. Finally, although all the patients who get referred to the MND Association receive the information material about the available financial support, assuming that all of them read the material and are equally able to make contact with the Association in order to claim financial support is unlikely. So, future research should also explore the extent to which the information about the services available by the Association is distributed equally and whether there are certain patients who are less likely to be aware of their entitlements.

Conclusions

The present results show that provision of financial support by the MND Association is provided across individuals and across boroughs regardless of their socio-economic circumstances. However, it seems that there is a difference across gender and age that benefits women and younger patients, respectively. Ethnic differences have not been

For peer review only

observed. Future research with a larger number of cases, spread across multiple regions, and more accurate data on the patients' socio-economic position and ethnic background would allow us to confirm these findings and draw more certain conclusions about the effectiveness of the MND Association's strategy in distributing financial support among patients fairly.

For peer review only

Acknowledgements

This research has been made possible thanks to a grant of the Motor Neurone Disease (MND) Association to Dr Valentina Gallo (grant number GPPG1G5R).

The funders had no role in influencing data analysis or result interpretation.

Dr Gallo had full access to all of the data in the study and takes responsibility for the integrity of the data and the accuracy of the data analysis. She declares that this manuscript is honest, accurate, and transparent account of the study being reported; that no important aspects of the study have been omitted. All co-authors had full access to the data (including statistical reports and tables), and can take responsibility for the integrity of the data and the accuracy of the data analysis.

Contributorship statement:

Study concept and design: V Gallo

Analysis and interpretation of data: V Gallo, A Giouleka, D Smith

Drafting of the manuscript: A Giuoleka

Data collection: A Manning

Critical revision of the manuscript for important intellectual content: A Malaspina, A Manning, D Smith, V Gallo

Conflict of interest statement:

We declare that we have no conflict of interests. The financial support from the MND Association has not influenced the submitted work.

Funding:

This article is part of the project “Social inequalities in accessing services provided by the MND Association” funded by the MND Association (GPPG1G5R)

Data sharing:

No additional unpublished data is available for this study.

For peer review only

Bibliography

1. MND Association available at https://www.mndassociation.org/2017).

2. van Es MA, Hardiman O, Chio A, et al. Amyotrophic lateral sclerosis. *Lancet* 2017.

3. Kiernan MC, Vucic S, Cheah BC, et al. Amyotrophic lateral sclerosis. *Lancet* 2011; **377**(9769): 942-55.

4. Chio A, Logroscino G, Traynor BJ, et al. Global epidemiology of amyotrophic lateral sclerosis: a systematic review of the published literature. *Neuroepidemiology* 2013; **41**(2): 118-30.

5. Watt G. The inverse care law today. *Lancet* 2002; **360**(9328): 252-4.

6. Wilkinson R, Marmot M. Social Determinants of Health - The solid facts. Denmark World Health Organisation 2003.

7. Arber S, Fenn K, Meadows R. Subjective financial well-being, income and health inequalities in mid and later life in Britain. *Soc Sci Med* 2014; **100**: 12-20.

8. Becares L. Which Ethnic groups have the poorest health? Ethnic identity and inequalities in Britain: the dynamics of diversity. Dynamics of diversity: evidence from the 2011 census University of manchester Joseph Rowntree Foundation 2015.

9. Nazroo JY, Williams DR, Marmot M, Wilkinson RG. The social determination of ethnic/racial inequalities in health Social Determinants of Health 2nd ed. Oxford Oxford University Press; 2006.

10. Sen A. Developments as freedom. Oxford Oxford paperbacks 2001.

11. Autority GL. Datastore LSOA Atlas - Greater London authority

12. Gill B. The English Indeces of Deprivation 2015: Departments for Communities and Local Government, 2015.

13. NOMIS. NOMIS - Official Labour Market Statistics.

14. O'Brien R, Hunt K, Hart G. 'It's caveman stuff, but that is to a certain extent how guys still operate': men's accounts of masculinity and help seeking. *Soc Sci Med* 2005; **61**(3): 503-16.

15. Noone JH, Stephens C. Men, masculine identities, and health care utilisation. *Sociol Health Illn* 2008; **30**(5): 711-25.

16. Sainsbury D. Gender, equality and welfare states. Cambridge: Cambridge University Press; 1996.

17. Scharf T. Multiple exclusion and quality of life amongst excluded older people in disadvantages neighborhoods. In: UK OotDPM-, editor. London; 2005.

18. Scharf T, Phillipson C, Smith AE. Social exclusion of older people in deprived urban communities of England. *Eur J Ageing* 2005; **2**(2): 76-87.

19. Dahlgren G, Whitehead M. Rainbow model of health. In: Europe WROf, ed. European Health Policy Conference: Opportunities for the Future Vol 11 –
Intersectoral Action for Health; 1991.

20. Link BG, Phelan J. Social conditions as fundamental causes of disease. *J Health Soc Behav* 1995; **Spec No**: 80-94.

21. Sui D. Ecological fallacy. In: Thrift N, ed. International Encyclopedia of Human Geography. Oxford: Elsevier; 2009: 291-3.

22. Deas, I., B. Robson, C. Wong and M. Bradford (2003). "Measuring neighbourhood deprivation: a critique of the Index of Multiple Deprivation." Environment and PlanningC: Government and Policy **21**: 883-903.

For peer review only

For peer review only

Table 1: Total population, number of people living with MND, mean, median age and standardised prevalence per 100,000 by gender and borough in greater London in 2016

			Men		Women	
**Borough**	**Total population**	**People with MND**	**Mean/median age**	**Standardised prevalence per 100,000**	**Mean/median age**	**Standardised prevalence per 100,000**
Barking and Dagenham	185,911	12	64/61	6.06	66/67	4.90
Barnet	356,386	22	64/66	7.04	77/74	2.16
Bexley	231,997	14	70/69	5.76	63/63	2.14
Brent	311,215	<10	43/43	1.21	67/67	3.06
Bromley	309,392	23	6365	2.33	70/68	4.89
Camden	220,338	13	62/65	3.41	52/60	4.07
Croydon	363,378	22	72/68	5.48	70/71	3.52
Ealing	338,449	15	53/52	6.08	75/72	1.69
Enfield	312,466	18	63/66	4.11	63/63	4.18
Greenwich	254,557	13	63/66	4.30	63/63	3.28
Hackney	246,270	<10	63/63	2.17	64/65	6.80
Hammersmith and Fulham	182,493	<10	68/68	3.71	60/60	3.25
Haringey	254,926	<10	59/59	1.74	40/40	0.65
Harrow	239,056	15	66/66	7.14	76/76	1.78
Havering	237,232	15	71/72	4.76	69/69	2.29
Hillingdon	273,936	14	6971	5.03	57/54	2.93
Hounslow	253,957	<10	62/62	2.80	50/43	2.80
Islington	206,125	<10	78/78	1.97		0.00
Kensington and Chelsea	158,649	<10	70/67	2.29	87/86	2.38
Kingston upon Thames	160,060	11	61/62	6.93	68/69	3.59
Lambeth	303,086	11	61/60	5.23	63/62	3.10
Lewisham	275,885	12	57/54	5.54	65/60	2.36
Merton	199,693	<10	67/73	4.77	63/51	2.06
Newham	307,984	11	60/60	7.93	63/63	0.82
Redbridge	278,970	24	67/66	7.93	74/79	4.93

For peer review only

Richmond upon Thames	186,990	11	72/74	3.13	71/72	4.36
Southwark	288,283	<10	65/70	4.13	79/79	1.18
Sutton	190,146	12	62/63	6.27	68/70	2.87
Tower Hamlets	254,096	<10	61/59	7.36	48/48	1.15
Waltham Forest	258,249	11	67/70	4.14	71/72	3.00
Wandsworth	306,995	13	72/73	4.36	69/74	3.80
Westminster	219,396	14	60/60	4.92	66/67	5.95
**Total**	**8,166,566**	**396**	**64 (SD 12)/ 65 (IQR 57-72)**	**5.13**	**68 (SD 13) 70 (IQR 61-76)**	**3.01**

SD=standard deviation; IQR = interquartile range

For peer review only

Table 2: General characteristics of the population living with MND in greater London, and divided by being recipient of a grant from MND Association

Characteristics	All N=396	Grant recipient N=96	Not grant recipient N=300	p-value*
Men (%)	233 (58.8)	49 (51.0)	184 (61.3)	0.075
Age, mean (SD)	65.7 (12.7)	65.0 (13.0)	66.0 (12.6)	0.523
Ethnicity				
White (%)	127 (32.1)	41 (32.3)	86 (67.7)	<0.001
Asian (%)	25 (6.3)	12 (48.0)	13 (52.0)	
Black (%)	13 (3.3)	5 (38.5)	8 (61.5)	
Mixed	7 (1.8)	2 (28.6)	5 (71.4)	
Other/unknown	224 (56.6)	188 (83.9)	36 (16.1)	
Index of Multiple Deprivation				
Decile, mean (SD)	5.4 (2.7)	5.5 (2.7)	5.4 (2.6)	0.773

* p-value from chi-square test for categorical variables, and t-test for continuous variables

For peer review only

Table 3: OR and relative 95% C.I> coming from logistic regression models investigating determinants of being recipient of an MND grant among prevalent cases of MND in greater London. Beta coefficients and relative 95% C.I. from linear regression models investigating factors associated with the amount of money awarded (in GBP, log transformed)

	All prevalent cases N=396		Grant recipients only N=96	
	**Logistic regression Crude OR (95% C.I)**	**Logistic regression Adjusted OR (95% C.I)**	**Linear regression Crude β coefficient (95% C.I)**	**Linear regression Adjusted β coefficient (95% C.I)**
Male gender	0.66 (0.41-1.04)	**0.60 (0.37-0.98)**	-0.078 (-0.58/0.42)	-0.11 (-0.62/0.39)
Age (years)	0.99 (0.98-1.01)	0.99 (0.98-1.01)	**-0.020 (-0.04/-0.001)**	**-0.02 (-0.04/-0.002)**
Ethnic group				
White	Ref.	Ref.	Ref.	Ref.
Black	1.31 (0.40-4.26)	1.32 (0.39-4.48)	0.52 (-0.65/1.69)	0.33 (-0.86/1.53)
Asian	1.94 (0.81-4.61)	2.18 (0.89-5.32)	-0.08 (-0.89/0.73)	-0.26 (-1.09/0.57)
Mixed	0.84 (0.16-4.51)	0.82 (0.15-4.49)	-0.09 (-1.88/1.69)	-0.30 (-2.09/1.49)
Other/unknown	**0.40 (0.24-0.67)**	**0.40 (0.24-0.68)**	0.17 (-0.39/0.73)	0.10 (-0.46/0.67)
IMD (deciles)	1.01 (0.93-1.10)	1.04 (0.95-1.14)	-0.005 (-0.097/0.087)	0.02 (-0.07/0.12)

For peer review only

Figure legends

Figure 1: Map of greater London showing the prevalence of MND by borough and gender

Figure 2: Boxplots representing total spent in GBP by the MND Association by ethnic group on Greater London (N=396)

Figure 3: Map of greater London showing prportion of people living with MND who received a grant from the MND Association, by borough, over the Index of Multiple Deprivation quintile

For peer review only

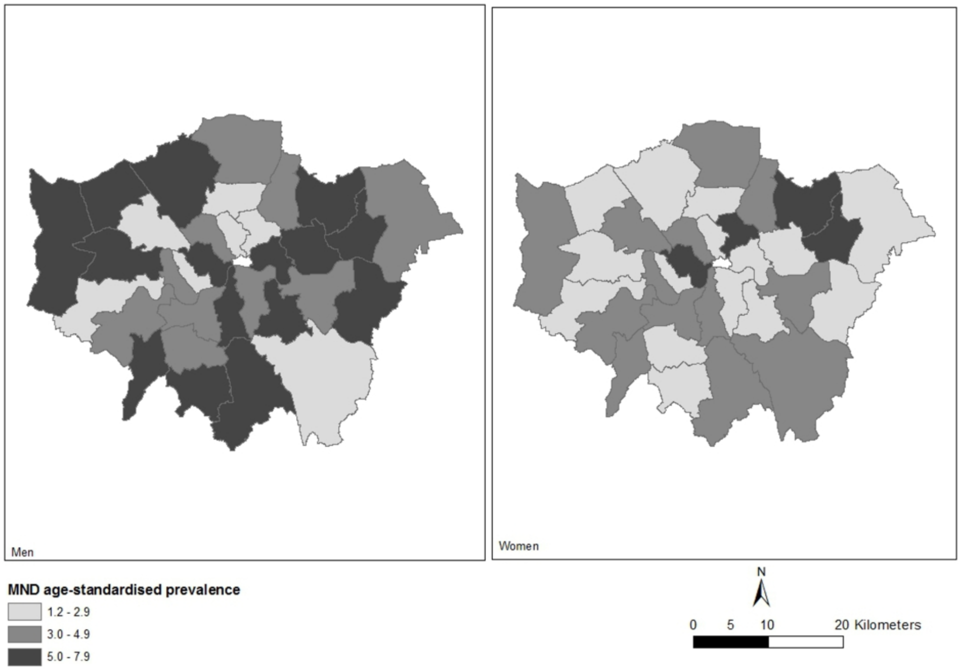

Figure 1: Map of greater London showing the prevalence of MND by borough and gender

205x145mm (300 x 300 DPI)

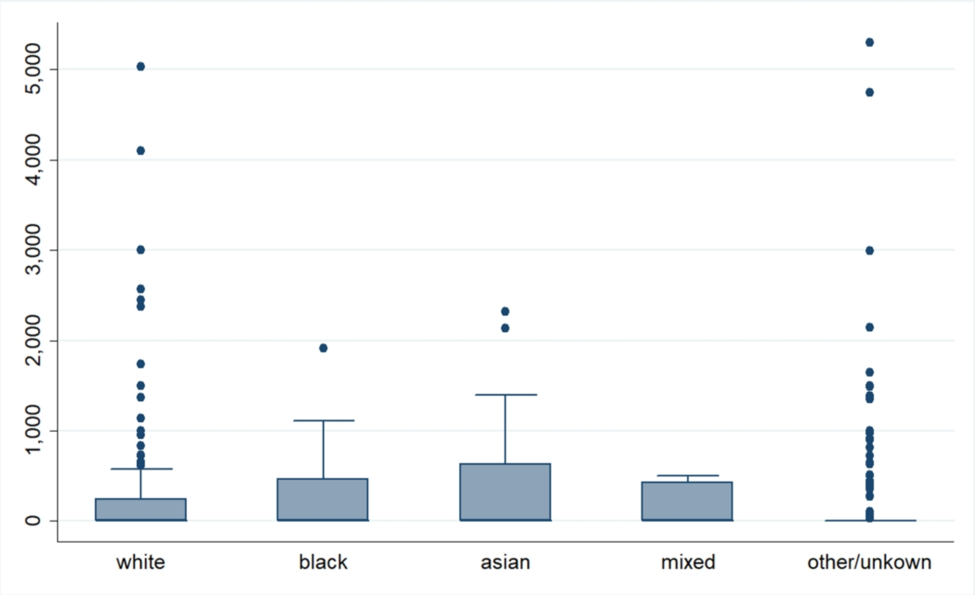

Figure 2: Boxplots representing total spent in GBP by the MND Association by ethnic group on Greater London (N=396)

205x131mm (300 x 300 DPI)

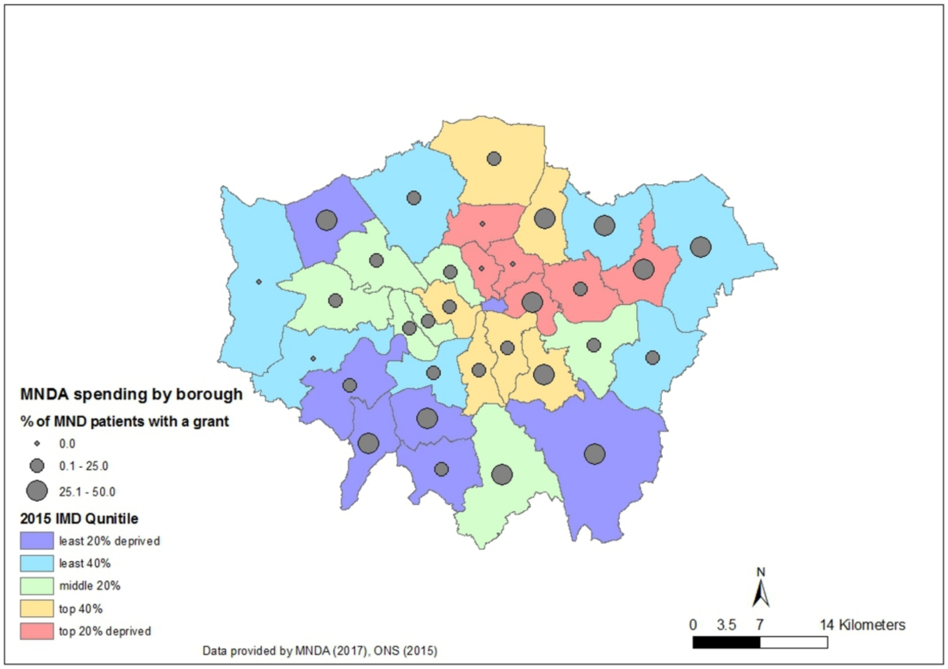

Figure 3: Map of greater London showing prportion of people living with MND who received a grant from the MND Association, by borough, over the Index of Multiple Deprivation quintile

207x146mm (300 x 300 DPI)

STROBE Statement—Checklist of items that should be included in reports of ***cross-sectional studies***

	Item No	Recommendation	Manuscript
**Title and abstract**	1	(*a*) Indicate the study's design with a commonly used term in the title or the abstract	N/A
		(*b*) Provide in the abstract an informative and balanced summary of what was done and what was found	p. 2
**Introduction**			
Background/rationale	2	Explain the scientific background and rationale for the investigation being reported	p. 5-6
Objectives	3	State specific objectives, including any prespecified hypotheses	p. 6
**Methods**			
Study design	4	Present key elements of study design early in the paper	p. 7
Setting	5	Describe the setting, locations, and relevant dates, including periods of recruitment, exposure, follow-up, and data collection	p. 6-7
Participants	6	(*a*) Give the eligibility criteria, and the sources and methods of selection of participants	p. 7
Variables	7	Clearly define all outcomes, exposures, predictors, potential confounders, and effect modifiers. Give diagnostic criteria, if applicable	p. 7
Data sources/ measurement	8*	For each variable of interest, give sources of data and details of methods of assessment (measurement). Describe comparability of assessment methods if there is more than one group	p. 7
Bias	9	Describe any efforts to address potential sources of bias	p. 7
Study size	10	Explain how the study size was arrived at	p. 6-7
Quantitative variables	11	Explain how quantitative variables were handled in the analyses. If applicable, describe which groupings were chosen and why	p. 7
Statistical methods	12	(*a*) Describe all statistical methods, including those used to control for confounding	p. 8
		(*b*) Describe any methods used to examine subgroups and interactions	N/A
		(*c*) Explain how missing data were addressed	p. 7
		(*d*) If applicable, describe analytical methods taking account of sampling strategy	N/A
		(*e*) Describe any sensitivity analyses	N/A
**Results**			
Participants	13*	(a) Report numbers of individuals at each stage of study—eg numbers potentially eligible, examined for eligibility, confirmed eligible, included in the study, completing follow-up, and analysed	p. 8
		(b) Give reasons for non-participation at each stage	N/A
		(c) Consider use of a flow diagram	N/A
Descriptive data	14*	(a) Give characteristics of study participants (eg demographic, clinical, social) and information on exposures and potential confounders	p. 8
		(b) Indicate number of participants with missing data for each variable of interest	p. 8

For peer review only

For peer review only - http://bmjopen.bmj.com/site/about/guidelines.xhtml

Outcome data	15*	Report numbers of outcome events or summary measures	p. 8
Main results	16	(*a*) Give unadjusted estimates and, if applicable, confounder-adjusted estimates and their precision (eg, 95% confidence interval). Make clear which confounders were adjusted for and why they were included	Table 2
		(*b*) Report category boundaries when continuous variables were categorized	N/A
		(*c*) If relevant, consider translating estimates of relative risk into absolute risk for a meaningful time period	N/A
Other analyses	17	Report other analyses done—eg analyses of subgroups and interactions, and sensitivity analyses	N/A
**Discussion**			
Key results	18	Summarise key results with reference to study objectives	p. 9-10
Limitations	19	Discuss limitations of the study, taking into account sources of potential bias or imprecision. Discuss both direction and magnitude of any potential bias	p. 12
Interpretation	20	Give a cautious overall interpretation of results considering objectives, limitations, multiplicity of analyses, results from similar studies, and other relevant evidence	p. 12
Generalisability	21	Discuss the generalisability (external validity) of the study results	p. 10-11
**Other information**			
Funding	22	Give the source of funding and the role of the funders for the present study and, if applicable, for the original study on which the present article is based	p. 13

*Give information separately for exposed and unexposed groups.

Note: An Explanation and Elaboration article discusses each checklist item and gives methodological background and published examples of transparent reporting. The STROBE checklist is best used in conjunction with this article (freely available on the Web sites of PLoS Medicine at http://www.plosmedicine.org/, Annals of Internal Medicine at http://www.annals.org/, and Epidemiology at http://www.epidem.com/). Information on the STROBE Initiative is available at www.strobe-statement.org.

For peer review only
